# QTL mapping and genome-wide prediction of heat tolerance in multiple connected populations of temperate maize

**DOI:** 10.1038/s41598-019-50853-2

**Published:** 2019-10-08

**Authors:** Delphine Van Inghelandt, Felix P. Frey, David Ries, Benjamin Stich

**Affiliations:** 1 Institute for Quantitative Genetics and Genomics of Plants, Cluster of Excellence on Plant Sciences (CEPLAS), Heinrich Heine University, 40225 Düsseldorf, Germany; 2 Max Planck Institute for Plant Breeding Research, 50829 Köln, Germany; 3 Present Address: Crop Functional Genomics, Institute of Crop Science and Resource Conservation, University of Bonn, Bonn, 53113 Germany

**Keywords:** Plant genetics, Plant breeding

## Abstract

Climate change will lead to increasing heat stress in the temperate regions of the world. The objectives of this study were the following: (I) to assess the phenotypic and genotypic diversity of traits related to heat tolerance of maize seedlings and dissect their genetic architecture by quantitative trait locus (QTL) mapping, (II) to compare the prediction ability of genome-wide prediction models using various numbers of KASP (Kompetitive Allele Specific PCR genotyping) single nucleotide polymorphisms (SNPs) and RAD (restriction site-associated DNA sequencing) SNPs, and (III) to examine the prediction ability of intra-, inter-, and mixed-pool calibrations. For the heat susceptibility index of five of the nine studied traits, we identified a total of six QTL, each explaining individually between 7 and 9% of the phenotypic variance. The prediction abilities observed for the genome-wide prediction models were high, especially for the within-population calibrations, and thus, the use of such approaches to select for heat tolerance at seedling stage is recommended. Furthermore, we have shown that for the traits examined in our study, populations created from inter-pool crosses are suitable training sets to predict populations derived from intra-pool crosses.

## Introduction

Maize (*Zea mays* L.), compared to other crop species which grow in temperate Europe, is heat tolerant due to its C4 metabolism and its tropical origin^1^. Nevertheless, temperate maize cultivars can experience substantial damages when encountering heat stress^2^. Especially during flowering and grain filling, heat stress has severe impacts on maize plants^3^. Phenotypic consequences of heat stress at adult stage on phenotypic traits of maize are, among others, a reduction of the time to flowering^4^, an increased anthesis-silking interval^5^, a reduction of photosynthetic tissue due to leaf scorching^4^, and a reduction of grain and whole plant yield^6^.

However, temperate maize is also significantly affected by heat events during seedling stage^7^. This is of particular practical importance for biogas production for which maize is the most important crop^8^ in temperate Europe. One of the cultivation practices is that the planting of biogas maize is postponed until the harvest of the winter cereals in early summer. With this cropping system, sensitive maize seedlings are exposed to heat stress^9^.

In the future, heat stress is expected to become an even more critical threat to crop cultivation in temperate regions than it is today^10^ as the mean temperature and the severity of heat events will rise due to climate change^11^. Therefore, breeding heat-tolerant cultivars is crucial to sustain crop production in the future^12^.

The tolerance to heat stress in maize was studied on a molecular level with a focus on natural variation by Ottaviano *et al*.^13^, Frova and Sari-Gorla^14^, and Reimer *et al*.^9^. These studies focused on isolated plant characteristics such as the cellular membrane stability, pollen germination, and root architecture. Alam *et al*.^5^ estimated variance components for traits involved in heat tolerance in field trials under natural heat stress condition. However, we are not aware of systematic studies characterising genetic variability for heat tolerance at seedling stage on a whole plant level.

The classical approach to improve a trait by breeding is to screen genetic material in one or several environments in which the conditions are such that the phenotypic trait of interest shows heritable variation. The issue with an evaluation of heat tolerance in natural environments is that the timing and the strength of the heat event are typically unpredictable^15^. One way to circumvent this problem is to screen the genetic material of interest in an artificial environment such as greenhouses or growth chambers. The efficiency of such approaches can be increased by combining them with marker-assisted selection approaches. For many years, the markers for such approaches have been identified by quantitative trait loci (QTL) mapping or genome-wide association mapping. Although numerous QTL have been identified for maize (for review see Sehgal *et al*.^16^), the impact of marker-assisted selection for improving truly quantitative traits in maize breeding is limited^17^. This is primarily attributed to the small effects of many of the detected QTL. An alternative promising approach for such traits is genome-wide prediction (GWP) because it captures not only the variance of the QTL but also all genetic variance. However, to the best of our knowledge, such an approach has not been tested previously for traits related to heat tolerance.

Several studies have shown that moderate-to-high genomic prediction accuracies can be obtained in bi-parental populations for a trait with high heritability, even by using low marker density and a relatively small training population^18–20^. Other studies have illustrated that genotypic characterisation using high-density genotyping platforms might improve the prediction accuracy^21–23^. To the best of our knowledge, only few experimental studies till now have compared GWP based on low density genotyping and genotyping by sequencing (GBS)^24–26^. Furthermore, the composition and size of the training and validation sets are crucial for GWP. Technow *et al*.^27^ analysed the possibility of combining training sets across heterotic pools. However, no earlier study has examined the suitability of segregating populations derived from inter-pool crosses as training set for the prediction of the two original heterotic pools.

The objectives of this study were the following: (I) to assess the phenotypic and genotypic diversity of traits related to heat tolerance of maize seedlings and dissect their genetic architecture by quantitative trait locus (QTL) mapping, (II) to compare the prediction ability of genome-wide prediction models using various numbers of KASP (Kompetitive Allele Specific PCR genotyping) single nucleotide polymorphisms (SNPs) and RAD (restriction site-associated DNA sequencing) SNPs, and (III) to examine the prediction ability of intra-, inter-, and mixed-pool calibrations.

## Methods

### Plant material and phenotypic evaluation

This study was based on six segregating populations derived from pairwise crosses of four Dent (S067 = *D*_1_, P040 = *D*_2_, S058 = *D*_3_, S070 = *D*_4_) and four Flint (L012 = *F*_1_, L017 = *F*_2_, L043 = *F*_3_, L023 = *F*_4_) maize inbred lines from the University of Hohenheim^28^. The eight inbred lines were previously characterised in detail for their heat tolerance at seedling stage^7^. The inbreds were crossed pairwise to create two Dent x Dent (DxD), two Flint x Flint (FxF), and two Dent x Flint (DxF) F_1_ genotypes (Supplementary Fig. 1). The F_1_ genotypes were further self-pollinated in an ear to row manner, resulting in six segregating populations (${{\rm{P}}}_{{D}_{1}{D}_{2}}$, ${{\rm{P}}}_{{D}_{3}{D}_{4}}$, ${{\rm{P}}}_{{F}_{1}{F}_{2}}$, ${{\rm{P}}}_{{F}_{3}{F}_{4}}$, ${{\rm{P}}}_{{D}_{1}{F}_{1}}$, ${{\rm{P}}}_{{D}_{4}{F}_{4}}$) comprising between 75 and 107 F_3:4_ progenies with a total of 607 genotypes.

Seeds were sown in soil (50% ED73, 50% Mini Tray (Einheitserde- und Humuswerke, Gebr. Patzer GmbH & Co. KG, Sinntal-Altengronau, Germany)) in single pots (9 cm edge length) as described previously. The experiment was replicated three times. The experimental design was a lattice design comprising 32 incomplete blocks per replication which were distributed on four tables in a walk-in growth chamber (Bronson Incubator Services B.V., Nieuwkuijk, Netherlands). The parental inbred lines were included as checks once on each table.

The plants were grown at 25 °C during a 16 h light period and at 20 °C during a 8 h dark period for a total of three weeks in the growth chamber; the relative humidity was set to 60% during this period. Photosynthetic active radiation emitted by fluorescent tubes was between 270–280 *μ*mol *m*^−2^ *s*^−1^ in the canopy of the plants. The plants were watered every morning with an automatic irrigation system (Itec DC station Multi Program, I.T. Systems Ltd., Bazra, Israel) to avoid drought stress.

The leaf growth rate was assessed as follows: fourteen days after sowing, the length of the fourth leaf from the shoot base to the leaf tip was measured daily for a period of three days during the stage of linear growth. The slope of a linear trend line of leaf length measurements vs. time represented the leaf growth rate (LR). Twenty days after sowing, leaf greenness (SD) (SPAD-502, Minolta Corporation, Ramsey, NJ, USA) was assessed as the maximum value of four readings on the leaf blade of the latest fully developed leaf. Furthermore, leaf scorching of young leaves (SC) and leaf senescence of old leaves (SN) were recorded on a scale of 1 (weak damage) to 9 (strong damage). The length of the fourth leaf (LL), the plant height (PH) from the shoot base to the point where the youngest leaf detached from the older leaf’s sheath, and the number of leaves (NL) with visible leaf ligule were recorded per plant. 21 days after sowing, shoot dry weight (DW) and the shoot water content (WC) of the fresh material were determined. The above outlined experiment was repeated at a higher heat level, where the temperature was increased, six days after planting, to 38 °C at day and 33 °C at night to induce heat stress for a two-week period. The study was, thus, based on two experiments with different heat levels, which will be referred to hereafter as standard and heat conditions.

### Genotyping

#### KASP SNPs

The parental inbred lines of the segregating populations were genotyped using a 50 K SNP array^29^. Out of 56,110 SNPs, 170 SNP markers were selected to genotype the individuals of the six segregating populations. For each population, between 47 and 77 markers were chosen (60 for ${{\rm{P}}}_{{D}_{1}{D}_{2}}$, 47 for ${{\rm{P}}}_{{D}_{3}{D}_{4}}$, 75 for ${{\rm{P}}}_{{F}_{1}{F}_{2}}$, 64 for ${{\rm{P}}}_{{F}_{3}{F}_{4}}$, 67 for ${{\rm{P}}}_{{D}_{1}{F}_{1}}$, and 77 for ${{\rm{P}}}_{{D}_{4}{F}_{4}}$) as they were polymorphic between the two parental inbreds of each population and showed no heterozygosity in any of the parental inbreds. SNP marker selection was optimised for equal distribution across the physical map (due to the unavailability of a genetic map at that time) and the overlapping of markers between populations. The selected SNP markers were genotyped using KASP SNP technology by TraitGenetics GmbH (Gatersleben, Germany) on a bulk of 6 to 10 F_3:4_ plants per genotype in the respective populations. This data set with 170 SNPs on 607 progenies will be designated hereafter as KASP_607_.

#### RAD SNPs

The RAD libraries of the segregating populations were prepared for single-end sequencing according to Baird *et al*.^30^ with the following modifications: barcodes were 5 bp long, and were at least two mutational steps apart from each other with regard to the first four bases, followed by the fifth checksum base. A total of 2 *μ*g genomic DNA of the same pool of plants that was used for KASP genotyping was digested for 30 min at 37 °C in a 50 *μ*l reaction with 20 units of *Kpn*I (New England Biolabs). Samples were inactivated by purification with Qiaquick spin columns (Qiagen, Hilden, Germany). Libraries were 96-fold barcoded, each genotype at two barcodes, resulting in 7 libraries. The sequencing of the RAD libraries was performed on a Hiseq2000 with 100 bp single end reads by the Max Planck-Genome-centre Cologne, Germany (https://mpgc.mpipz.mpg.de/home/), following the manufacturer’s protocol.

Demultiplexing of raw sequencing data by barcode was performed using the Stacks software pipeline^31^. Parameters were chosen to allow barcode rescue with a distance of two, where reads were discarded according to default settings. In the next steps, version 0.4.2 of Trim Galore! (http://www.bioinformatics.babraham.ac.uk/projects/trim_galore/) was used for adapter and quality trimming. In addition to the standard parameters, reads were filtered for a length of at least 75 bp, and the threshold for trimming low quality ends from reads was increased to 30 for higher stringency.

The trimmed reads were mapped genotype-wise to the repeat masked and concatenated reference sequence, using BWA-MEM. In accordance to previous studies^32^, apart from an increased sensitivity parameter of -r 1.0, standard parameters were used.

For genotype calling, GATK’s HaplotypeCaller^33^ was applied to each genotype’s bam file independently, where the allowed maximum number of alternative alleles was set to three. The minimum base and mapping quality for calling were set to 30 and 40, respectively. Finally, soft-clipped bases were excluded from the analysis. All other parameters were set to their default values. Finally, the resulting files were combined into one and each position was recalculated and re-genotyped, considering information of all samples, by GATK’s genotypeGVCFs. To ensure a high quality of the genotype calls, many filtering steps (see SM1) were applied to the resulting genotype call file.

After genotype calling, 53,579 SNP loci remained on 489 genotypes comprising 482 progenies and 7 parental inbred lines (Supplementary Fig. 1). Missing genotype calls were imputed per population using Beagle^34^. Three cutoff values were chosen to filter per genotype the genotype calls based on the genotype probabilities (GP). For each of the three GP cutoff values, one of the three possible genotypes calls (minor allele homozygous, heterozygous, and major allele homozygous) was assigned to a genotype only when its predicted probability was greater than X for a given imputed genotype call, and all others were set to “NA”. The three cutoff values were X = 1, 0.98^35^, and the allele with maximum genotype probability (MAX). The such created missing genotype calls were mean imputed. The data sets associated with the three cutoff values will be designated hereafter as RAD_482-GP:1_, RAD_482-GP:0.98_, and RAD_482-GP:MAX_, respectively. As there were less genotypes in the RAD than in the KASP data set, we defined an additional KASP data set with the same 482 progenies as for the RAD data set. This will be referred to hereafter as KASP_482_.

#### Diversity

Gene diversity D^36^ and population differentiation G_ST_^37^ were calculated. The average modified Roger’s distances (MRD) between and within populations were calculated according to Wright *et al*.^38^. Associations among genotypes were revealed with a principal component analysis (PCA) based on MRD estimates.

### Phenotypic data analysis

#### Adjusted entry mean calculation

To assess the significance of the heat level effect, the following model was used:

1 $${Y}_{icrb}=\mu +{G}_{i}+{C}_{c}+{(G.C)}_{ic}+{R}_{cr}+{B}_{crb}+{e}_{icrb},$$

where *Y*_*icrb*_ is the phenotypic observation of the *i*^*th*^ genotype in the *b*^*th*^ block within the *r*^*th*^ replication nested within the *c*^*th*^ heat level. *μ* is the general mean, *G*_*i*_ is the effect of the *i*^*th*^ genotype, *C*_*c*_ is the effect of the *c*^*th*^ heat level, ${(C.G)}_{ci}$ is the interaction between the *i*^*th*^ genotype and the *c*^*th*^ heat level, *R*_*cr*_ is the effect of the *r*^*th*^ replication nested within the *c*^*th*^ heat level, *B*_*crb*_ is the effect of the *b*^*th*^ block in the *r*^*th*^ replication nested within the *c*^*th*^ heat level and *e*_*icrb*_ the residual. *C*_*c*_ was regarded as fixed and all other effects were set as random. Traits with a significant *C*_*c*_ effect were considered heat-dependent traits. The adjusted entry means (AEM) of standard and heat conditions across genotypes were estimated and will be refered to as AEM_s_ and AEM_H_, respectively.

To calculate AEM for each assessed trait of each genotype in each condition (AEM_*i*S_ and AEM_*i*H_ for standard and heat condition, respectively), the phenotypic observations of each heat level were separately analysed using the following model:

2 $${Y}_{irb}=\mu +{G}_{i}+{R}_{r}+{B}_{rb}+{e}_{irb},$$

where *Y*_*irb*_ is the phenotypic observation of the *i*^*th*^ genotype in the *r*^*th*^ replication and the *b*^*th*^ block, *R*_*r*_ is the effect of the *r*^*th*^ replication, *B*_*rb*_ is the effect of the *b*^*th*^ block nested within the *r*^*th*^ replication, and *e*_*irb*_ the residual. The genotype effect *G*_*i*_ was of primary interest in this analysis and was considered a fixed effect. *R*_*r*_ was considered fixed and *B*_*rb*_ random.

#### Heat susceptibility index

A heat susceptibility index (HSI) for each trait and each genotype was calculated according to Mason *et al*.^39^:

3 $${{\rm{HSI}}}_{i}=\frac{1-{{\rm{AEM}}}_{i{\rm{H}}}/{{\rm{AEM}}}_{i{\rm{S}}}}{1-{{\rm{AEM}}}_{{\rm{H}}}/{{\rm{AEM}}}_{{\rm{S}}}},$$

where HSI_*i*_ is the HSI for genotype *i*. The HSI of the individual traits will be designated as follows: HSI_LL_, HSI_PH_, HSI_NL_, HSI_SC_, HSI_SN_, HSI_SD_, HSI_DW_, HSI_WC_, and HSI_LR_. Pairwise Pearson correlation coefficients were calculated between the HSI of all assessed traits across all genotypes. Further, HSI of this study were correlated with HSI assessed during adult stage under field conditions^4^.

#### Variance components and heritability

Genotypic (${\sigma }_{g}^{2}$) and error (${\sigma }_{e}^{2}$) variance components for each heat level were calculated using model (2) with a random *G*_*i*_ effect. For each trait, the broad sense heritability (h^2,^^40^) of the observations at each heat level was calculated separately for each (${{\rm{h}}}_{{\rm{pop}}}^{{\rm{2}}}$) as well as across (${{\rm{h}}}_{{\rm{a}}}^{{\rm{2}}}$) the six populations. Heritability can not be assessed for HSI directly as it is not measured but calculated on a mean basis with no replication^39^. To calculate an approximate heritability for the HSI of each trait, we applied the following procedure, which is an extension of the approach of Ouk *et al*.^41^. For each of the two heat levels, we estimated, based on model (2), the fixed effects for *R*_*r*_ and *B*_*rb*_. In a second step, these effects were subtracted from all observations from the corresponding replicate and block to correct for differences in replicate and block effects. The adjusted data thereby comprised three replications per genotype for each of the two conditions. Subsequently, we separately created random pairs for each genotype between one replication from the heat condition and one from the standard condition. Across all genotypes, ${3}^{{2}^{607}}$ combinations of replications from the heat and standard conditions are possible. In the next step, for one possible combination of replications, the HSI was calculated as described previously, resulting in three HSI replicates for each genotype. These data were then analysed using the following model:

4 $${Y}_{ir}=\mu +{G}_{i}+{e}_{ir},$$

where *Y*_*ir*_ is the phenotypic observation of the *i*^*th*^ genotype in the *r*^*th*^ replication, *μ* is the general mean, *G*_*i*_ is the effect of the *i*^*th*^ genotype, and *e*_*ir*_ the residual. Based on genotypic (${\sigma }_{g}^{2}$) and error (${\sigma }_{e}^{2}$) variance components across all populations and for each population, the heritability was calculated. This procedure was repeated 100 times, and the median of the heritability was designed as heritability of HSI (${{\rm{h}}}_{{\rm{HSI}}}^{{\rm{2}}}$).

The genetic variance among and within populations was estimated for each HSI by partitioning the genotype effect of model (4) into the effect of the population and that of the genotype nested within the population. This procedure was repeated 100 times with random assignments between the different replications as explained for the ${{\rm{h}}}_{{\rm{HSI}}}^{{\rm{2}}}$ calculation, and the median of the genetic variance among and within populations was calculated. The genetic differentiation observed for the HSI was then calculated^42–44^ and will be designed hereafter as Q_ST_.

### QTL analyses with KASP SNPs

SNP markers with a significant (P < 0.001) observed deviation from the expected allele frequency were excluded from the analysis (cf. Benke *et al*.^45^). To improve the mapping of markers, information of five additional segregating populations which had been genotyped with the same set of SNP markers by Horn *et al*.^46^ were considered during map creation. A consensus genetic linkage map was calculated chromosome-wise using the software CarthaGène^47^.

QTL associated with heat tolerance were detected based on the consensus genetic linkage map using an iterative composite interval mapping approach^48^, implemented in the software MCQTL^49,50^. QTL analyses were performed for the HSI of the individual traits across all six populations. We took into account connections between populations using an additivve kinship matrix. As the studied bi-parental F_3:4_ populations have an expected heterozygosity of 25%, dominance effects between parental alleles of each bi-parental population were considered in the QTL analysis. Genotypic probabilities were computed every 5 centiMorgan (cM), taking into account information from neighboring markers. F thresholds to detect QTL for each trait were determined by 1 000 permutations, to adhere to a global type I error of 5% across populations and the entire genome^51^. F thresholds used to select co-factors were fixed at 90% of the F threshold values for QTL detection.

SNP markers associated with the respective HSI were selected as co-factors by forward regression, where the minimal distance between two co-factors was 10 cM. At the end of the detection process, confidence intervals were estimated on the basis of a 1.5 LOD unit fall^52^.

The dominance effect of each QTL was tested for its significance (P < 0.05) in each population using a two-sided t-test. The difference between the additive effects of pairs of parental alleles on the respective QTL was tested a posteriori using a Tukey test.

Furthermore, we overlapped the QTL detected in this study with those identified in a companion study at adult stage^4^. To identify candidate genes for heat tolerance in terms of the assessed traits, we mined genes, identified by Frey *et al*.^7^ as heat responsive or heat tolerance genes. We determined the position of these genes on the genetic map by linear regression with information of the nearest two SNP markers. Heat tolerance genes of Frey *et al*.^7^ mapping to QTL confidence intervals were designated in the following as heat tolerance candidate genes.

### GWP

#### Genetic model

GWP was performed by genomic best linear unbiased prediction (GBLUP^53^):

5 $$y={\bf{X}}\beta +{\bf{Z}}u+e,$$

where *y* is the vector of the HSI of the corresponding trait, **X** is a design matrix assigning fixed effects to the genotypes, *β* is a vector of fixed effects, **Z** is the design matrix that assigned the random effects to the genotypes, and *u* the vector of the random effects that were assumed to be normally distributed with: $u\sim N(0,{\bf{K}}{\sigma }_{u}^{2})$, where **K** denotes the realised kinship matrix calculated on the basis of the molecular marker data^54^ with ${\sigma }_{u}^{2}$ being the variance pertaining to the GBLUP model. Residuals *e* were assumed to be independent and normally distributed with $e\sim N(0,{\bf{I}}{\sigma }^{2})$, where **I** is the identity matrix and *σ*^2^ the residual variance.

Two different genetic models were examined for each type of molecular marker that differed in the **K** matrix used: M_*A*_ considering only additive effects with **K**_*A*_ (the additive kinship matrix) and M_*AD*_ considering additive and dominance effects with **K**_*A*_ and **K**_*D*_ (the dominance kinship matrix)^55^. GBLUP method was used as implemented in the R package Sommer^56^.

#### Model training and performance assessment

The effects were estimated in a training set (TS) and used in a second step to predict the breeding values of the genotypes of a validation set (VS). As measure of model performance, prediction ability was calculated as the Pearson correlation coefficient between observed and predicted phenotypes of the VS, $r(y,\hat{y})$^57^. In our study, different TS were used to establish the prediction model: in the TS_*pop*_ scenario, the model was trained using one or two populations; for the TS_*a*_ scenario all populations were used together as TS. For later scenario, prediction abilities were calculated both across all populations (*r*_*a*_) as well as for each population (*r*_*pop*_).

#### Resampling schemes

**Figure 1:**
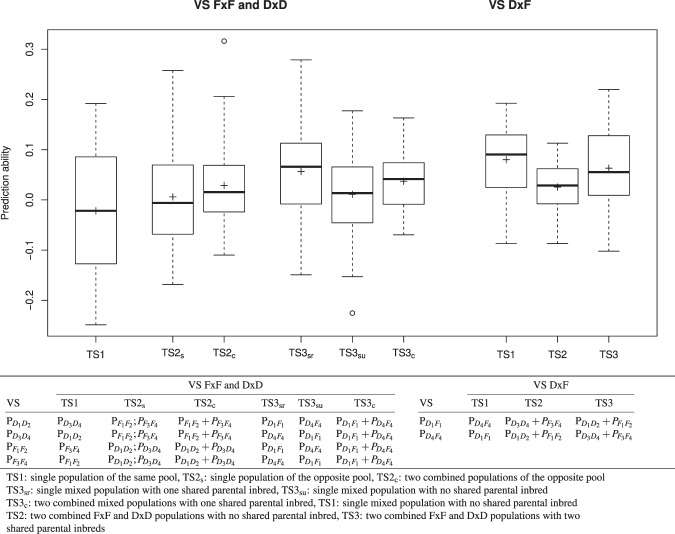
Boxplot of the between-population prediction abilities ${r}_{pop,BS{P}_{b}}$ across nine heat susceptibility indexes (HSI) and different validation sets (VS) using a boostrapping procedure with 50 genotypes for three different types of training set (TS) compositions. The analyses were based on RAD_482-GP:0.98_ and the M_*A*_ model.

Different types of resampling schemes were used to evaluate the model performance as a function of the examined TS*VS combination:

- For TS_*a*_, we performed 20 replications of five-fold cross validation (CV) to assess the model performance across all populations. Accordingly, the entire data set was subdivided into five mutually independent subsets where four formed the training set (TS) and one the validation set (VS). To account for the structure of our data set with six different segregating populations, the proportion of genotypes from the different segregating populations in the individual subsets was kept identical to that observed in the entire data set. The median of the Pearson correlation coefficient between observed and predicted phenotypes of the validation set $r({y}_{VS},{\hat{y}}_{VS})$ was used to assess model performance. Prediction abilities were calculated both across all populations (*r*_*a*,*cv*_) as well as for each population (*r*_*pop*,*cv*_).
- In the context of prediction within populations with TS_*pop*_, the influence of the size of the training set on the prediction ability was analysed by simulating, for each segregating population, a TS size *N* from 10 to 100 in steps of 10 using a bootstrapping approach. The median of the prediction ability across 500 replications was calculated for the HSI of each trait, genetic model, and type of SNP marker within each population and is designated in the following as ${r}_{pop,BS{P}_{w}}$.
- For the between population prediction using TS_*pop*_, the influence of the composition of the TS on the prediction ability was examined using three primary scenarios of TS compositions TS1, TS2, and TS3. In case of using the FxF and DxD populations as VS, TS1 comprised genotypes from populations for which the parental genotypes were from the same heterotic pool as those of the VS. TS2 comprised genotypes from one (TS2_s_) or two (TS2_c_) populations in which the parental genotypes belonged to the opposite heterotic pool as those of the VS. TS3 comprised genotypes from the DxF populations with three different scenarios: TS3_sr_ used the related mixed population, TS3_su_ used the unrelated mixed population, and TS3_c_ combined both mixed populations in the TS (Fig. 1, bottom).

In case of using the DxF populations as VS, the three scenarios were selected accordingly: TS1 comprised genotypes of the other DxF segregating population; TS2 comprised genotypes of the two combined FxF and DxD populations which did not have a parental inbred in common with the population in the VS. TS3 comprised genotypes of the two combined FxF and DxD populations which had a parental inbred in common with the population in the VS. To avoid differences in the prediction ability due to sample size effects, the TS were randomly reduced to a size of 50 such that the structure of the original TS was maintained. To obtain stable estimates for the prediction ability, the median of 500 independent bootstrapping runs of the TS construction was calculated for the HSI of each trait, genetic model, type of molecular marker, and TS*VS combination. The prediction abilities based on this bootstrapping strategy between populations will be referred to hereafter as ${r}_{pop,BS{P}_{b}}$.

#### Number of molecular markers

In order to examine the influence of the number of molecular markers on the prediction ability, X random RAD SNPs were sampled from the RAD_482-GP:0.98_, where X ranged from 170 to 43 520 in steps of $2\,\ast \,X$. Based on the selected SNPs, genomic predictions were obtained for the HSI of each traits using the M_*A*_ genetic model, and the median of the prediction abilities across 100 replications was calculated.

#### Comparison between observed and expected within-population prediction ability

We used the formula suggested by Daetwyler *et al*.^58^ to estimate the expected within-population prediction ability. Following Meuwissen *et al*.^59^, *M*_*e*_ was estimated as ${M}_{e}=2{N}_{e}L$ where L is the genome size in Morgans. L = 22.36 was adopted from a previous linkage-mapping study, using an F_2_ population which was characterised by genotyping by sequencing^60^ similarly to our study. The effective population size (*N*_*e*_) was calculated using the harmonic mean approximation for two generations (Hartl and Clark, p. 291^61^), resulting in Ne = 2.92, 2.93, 2.94, 2.93, 2.92, and 2.92 for populations ${{\rm{P}}}_{{D}_{1}{D}_{2}}$, ${{\rm{P}}}_{{D}_{3}{D}_{4}}$, ${{\rm{P}}}_{{F}_{1}{F}_{2}}$, ${{\rm{P}}}_{{F}_{3}{F}_{4}}$, ${{\rm{P}}}_{{D}_{1}{F}_{1}}$, and ${{\rm{P}}}_{{D}_{4}{F}_{4}}$, respectively.

## Results

### Phenotypic diversity

The condition effect (standard vs. heat) was significant ($P < 0.01$) across all populations and for all traits. Across the two examined conditions, ${{\rm{h}}}_{{\rm{a}}}^{{\rm{2}}}$ was medium to high (0.50–0.83) for all assessed traits (Table 1). For all traits except NL and SD, ${{\rm{h}}}_{{\rm{a}}}^{{\rm{2}}}$ was higher at heat compared to standard condition. ${{\rm{h}}}_{{\rm{HSI}}}^{{\rm{2}}}$ ranged from 0.41 (NL) to 0.75 (SC) and was with the exception of SC, WC, and LR lower than those of the traits in the two conditions. The heritability values observed on a population level (${{\rm{h}}}_{{\rm{pop}}}^{{\rm{2}}}$) were very low for a few trait*population*condition levels (Supplementary Table 1). This effect was the strongest for SC for which, at standard condition, a ${{\rm{h}}}_{{\rm{pop}}}^{{\rm{2}}}$ of 0 was observed for four of the six populations and smaller than 0.25 for the remaining two populations.

**Table 1:** Mean and range of the adjusted entry mean (AEM), broad sense heritability (${{\rm{h}}}_{{\rm{a}}}^{{\rm{2}}}$) of the studied traits for each condition, as well as for the heat susceptibility index (${{\rm{h}}}_{{\rm{HSI}}}^{{\rm{2}}}$), significance of the environmental condition (standard vs heat) effect for each trait, and degree of genetic differentiation among the six populations (Q_ST_).

Trait	Standard condition (25 °C)			Heat condition (38 °C)			HIS ${{\bf{h}}}_{{\bf{H}}{\bf{S}}{\bf{I}}}^{{\bf{2}}}$	Condition effect	Q_ST_
	Mean (AEM)	Range (AEM)	${{\bf{h}}}_{{\bf{a}}}^{{\bf{2}}}$	Mean (AEM)	Range (AEM)	${{\bf{h}}}_{{\bf{a}}}^{{\bf{2}}}$			
Leaf length	59.69	35.40–84.20	0.73	41.90	12.38–59.00	0.79	0.56	***	0.18
Plant height	18.52	9.45–24.18	0.58	12.99	2.86–20.00	0.73	0.52	**	0.16
Number of leaves	3.47	2.44–4.35	0.71	4.48	2.41–6.01	0.63	0.41	***	0.23
Leaf scorching	1.05	1.00–6.34	0.49	3.51	0.78–9.18	0.82	0.75	***	0.37
Leaf senescence	1.80	0.75–6.60	0.57	3.36	1.07–8.38	0.70	0.50	***	0.07
Leaf greenness	50.09	37.38–63.87	0.83	38.79	23.44–53.44	0.67	0.64	***	0.25
Shoot dry weight	0.91	0.31–1.54	0.69	0.70	0.12–1.64	0.76	0.67	***	0.17
Shoot water content	0.93	0.90–0.95	0.54	0.89	0.81–0.93	0.59	0.56	***	0.26
Leaf growth rate	0.25	0.12–0.39	0.50	0.22	−0.01–0.37	0.70	0.54	***	0.17

*, **, *** Significant at the 0.05, 0.01, and 0.001 probability level, respectively.

For LL, SC, DW, and LR, the mean and variation of the HSI were significantly ($P < 0.05$) higher in the two DxD populations (${{\rm{P}}}_{{D}_{1}{D}_{2}}$ and ${{\rm{P}}}_{{D}_{3}{D}_{4}}$) than in the FxF and DxF populations. Especially, the population ${{\rm{P}}}_{{D}_{3}{D}_{4}}$ differed significantly from the other populations in the mean HSI for all traits except SD (Supplementary Fig. 2). Q_ST_ calculated for the HSI varied from 0.07 (SN) to 0.37 (SC; Table 1).

The first two principal components (PC) of the PCA of the HSI explained 45% and 14% of the total variance, respectively (Supplementary Fig. 3). PC1 was significantly (${\rm{P}} < 0.01$) correlated with each HSI where the correlation coefficient was low (<|0.25|) for HSI_SD_ and between 0.36 and 0.85 for the other HSI (Supplementary Fig. 4). PC2 was significantly correlated with the HSI of each trait except HSI_NL_ and HSI_WC_ where the correlation coefficient was low (<|0.25|) for all HSI except for HSI_SC_ and HSI_SD_. The cluster of the two DxD populations overlapped only weakly with the cluster of the two FxF populations (Supplementary Fig. 3).

### Genetic diversity

The consensus genetic linkage map for KASP SNPs had a total length of 1 823.5 cM with an average distance of 11.3 cM and a maximum distance of 83.2 cM between two adjacent markers.

In the PCA based on MRD estimates calculated from KASP_607_, the first and second PC explained 21.87% and 11.87% of the molecular variance, respectively (Supplementary Fig. 5). For RAD_482-GP:0.98_, PC1 and PC2 explained 25.94 and 13.50% of the molecular variance. In the PCA based on KASP_607_, five distinct clusters were observed where one cluster was always constituted by the individuals of one segregating population except the individuals of the two FxF populations which were located in one overlapping cluster. For the RAD SNPs, the same trend was observed, but the two FxF populations (${{\rm{P}}}_{{F}_{1}{F}_{2}}$ and ${{\rm{P}}}_{{F}_{3}{F}_{4}}$) were assigned to two distinct clusters.

The lowest gene diversity was observed for ${{\rm{P}}}_{{D}_{3}{D}_{4}}$ irrespective of the considered marker type (Supplementary Table 2). The ranking between the populations for D and Gst was different when calculated based on KASP or RAD SNPs. The correlation between the MRD distance matrices calculated with KASP and RAD SNPs was with 0.84 significantly ($P=1.67\times {10}^{-4}$) different from 0. The correlation between the **K**_*A*_ matrices calculated with KASP and RAD SNPs was with 0.56 considerably lower but also significantly ($P=6\times {10}^{-4}$) different from 0.

### QTL mapping and overlapping region with QTL detected at adult stage

**Figure 2:**
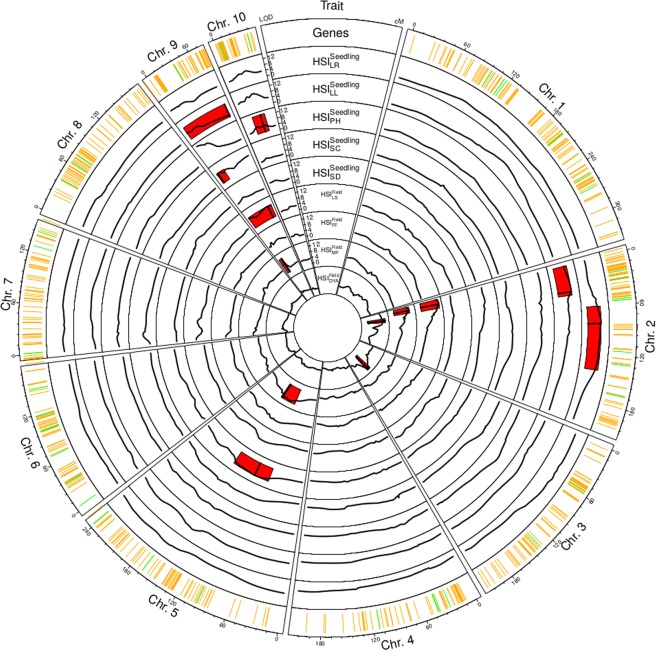
Circle plot showing the location of quantitative trait loci (QTL) affecting heat tolerance of maize. Heat tolerance (green) and heat responsive (orange) candidate genes^7^ are represented in the first track. Tracks 2–10 show logarithmic odds ratio (LOD) scores (black), detected QTL and confidence intervals (red) of the QTL for the heat susceptibility indexes (HSI) of the traits: leaf elongation rate (LR), leaf length (LL), plant height (PH), leaf scorching (SC) and leaf greenness (SD) at seedling stage, and leaf scorching (LS), time to female (FF) and male flowering (MF) and adjusted dry yield (DYA) at adult stage^4^.

We identified a total of six QTL for the HSI of five of the nine traits (Table 2), each explaining between 7% and 9% of the phenotypic variance (R^2^). The detected QTL were not randomly distributed across the genome, but a total of three QTL hot spots were observed. The QTL detected by Frey *et al*.^4^ for adult traits in field trials colocated with these hotspots (Fig. 2). Five of the heat tolerance genes identified by Frey *et al*.^7^ were located within the six QTL confidence intervals detected in our study (Supplementary Table 3).

**Table 2:** Quantitative trait loci (QTL) detected for the heat susceptibility index (HSI) of five traits (Leaf length: LL, Plant height: PH, Leaf scorching: SC, Leaf greenness: SD, Leaf growth rate: LR) at a significance level of ${\rm{P}} < 0.05$, with genetic map position [cM], logarithmic odds ratio (LOD) and their support interval, proportion of explained phenotypic variance (R^2^), additive effects of each parental genotype and dominance effects of the six populations.

Trait	QTL	Chr	Pos	LOD	Interval	R^2^	Additive effect of parent								Dominance effect of population					
							*D*_1_	*D*_2_	*D*_3_	*D*_4_	*F*_1_	*F*_2_	*F*_3_	*F*_4_	${{\bf{P}}}_{{{\boldsymbol{D}}}_{{\bf{1}}}{{\boldsymbol{D}}}_{{\bf{2}}}}$	${{\bf{P}}}_{{{\boldsymbol{D}}}_{{\bf{3}}}{{\boldsymbol{D}}}_{{\bf{4}}}}$	${{\bf{P}}}_{{{\boldsymbol{F}}}_{{\bf{1}}}{{\boldsymbol{F}}}_{{\bf{2}}}}$	${{\bf{P}}}_{{{\boldsymbol{F}}}_{{\bf{3}}}{{\boldsymbol{F}}}_{{\bf{4}}}}$	${{\bf{P}}}_{{{\boldsymbol{D}}}_{{\bf{1}}}{{\boldsymbol{F}}}_{{\bf{1}}}}$	${{\bf{P}}}_{{{\boldsymbol{D}}}_{{\bf{4}}}{{\boldsymbol{F}}}_{{\bf{4}}}}$
HSI_LL_	Q_HSI:LLa_	2	37	9.64	0–42	0.07	0.08^B^	−0.11^C^	0.07^AB^	−0.06^AC^	0.02^ABC^	0.01^ABC^	0.03^ABC^	−0.04^AC^	−0.00^ns^	−0.23^ns^	−0.02^ns^	−4.40^ns^	−0.03^ns^	0.78^ns^
	Q_HSI:LLb_	10	30	10.55	14–38	0.07	−0.00^AB^	−0.05^AB^	−0.03^AB^	−0.10^B^	0.03^A^	0.02^AB^	0.06^AB^	0.07^A^	0.00^ns^	−0.06^ns^	−0.19**	0.05^ns^	−0.07^ns^	0.29^ns^
	Simultaneous fit					0.13														
HSI_PH_	Q_HSI:PH_	9	3	13.20	0–17	0.09	−0.11^BD^	0.01^ABCD^	0.10^A^	−0.13^CD^	0.05^A^	0.05^AB^	0.01^ABCD^	0.03^AC^	0.10^ns^	−0.00^ns^	0.12^ns^	0.20^ns^	0.05^ns^	0.15^ns^
HSI_SC_	Q_HSI:SC_	9	74	8.88	0–74	0.07	−0.33^A^	0.50^A^	−0.02^A^	0.03^A^	−0.35^A^	0.18^A^	−0.02^A^	0.01^A^	1.34***	1.05**	−1.19^ns^	0.29^ns^	−2.51^ns^	−0.28^ns^
HSI_SD_	Q_HSI:SD_	5	101	10.06	70–151	0.08	0.10^B^	−0.27^A^	−0.22^A^	−0.23^A^	−0.01^AB^	0.18^B^	0.26^B^	0.19^B^	−0.17^ns^	0.13^ns^	−0.16^ns^	−0.58^ns^	−0.00^ns^	−0.11^ns^
HSI_LR_	Q_HSI:LR_	2	82	10.06	60–141	0.07	−0.24^AB^	−1.49^C^	0.12^AB^	−0.06^AB^	1.00^B^	0.73^AB^	0.13^AB^	−0.19^A^	0.99^ns^	−3.63^ns^	0.24^ns^	−0.69^ns^	−0.87^ns^	0.66^ns^

A, B, C, D Additive effects of parental inbreds with the same letters are not significantly (${\rm{P}} < 0.05$) different from each other.

*, **, *** Significant at the 0.05, 0.01 and 0.001 probability level, respectively.

^ns^Not significant.

### GWP

The square root of the proportion of phenotypic variance explained by the QTL for the HSI of all traits was lower than the prediction abilities of the GWP models, irrespective of the type of molecular marker and genetic model used (Table 3). Furthermore, the correlation between R_*QTL*_ and *r*_*a*_ across the nine examined traits for KASP_607_ was approximately 0.33.

**Table 3:** Square root of the proportion of the explained phenotypic variance of the QTL (R_QTL_), genome-wide prediction ability based on the different KASP and RAD SNP sets for the heat susceptibility index (HSI) of nine traits (leaf length (LL), plant height (PH), number of leaves (NL), leaf scorching (SC), leaf senescence (SN), leaf greenness (SD), shoot dry weight (DW), shoot water content (WC) and leaf growth rate (LR)) across the six populations (*r*_*a*_) using the additive genetic model M_*A*_.

Data set	R_QTL_	*r*_*a*_	*r*_*a*_	*r*_*a*_	*r*_*a*_	*r*_*a*_
	KASP_607_	KASP_607_	KASP_482_	RAD_482-GP:MAX_	RAD_482-GP:0.98_	RAD_482-GP:1_
Trait						
HSI_LL_	0.36	0.66	0.64	0.88	0.92	0.93
HSI_PH_	0.30	0.64	0.63	0.88	0.94	0.94
HSI_NL_	0.00	0.58	0.58	0.69	0.70	0.70
HSI_SC_	0.26	0.80	0.81	0.85	0.88	0.88
HSI_SN_	0.00	0.56	0.52	0.68	0.71	0.76
HSI_SD_	0.28	0.69	0.66	0.80	0.90	0.83
HSI_DW_	0.00	0.63	0.62	0.88	0.93	0.94
HSI_WC_	0.00	0.65	0.68	0.89	0.88	0.86
HSI_LR_	0.26	0.71	0.70	0.89	0.92	0.93

**Figure 3:**
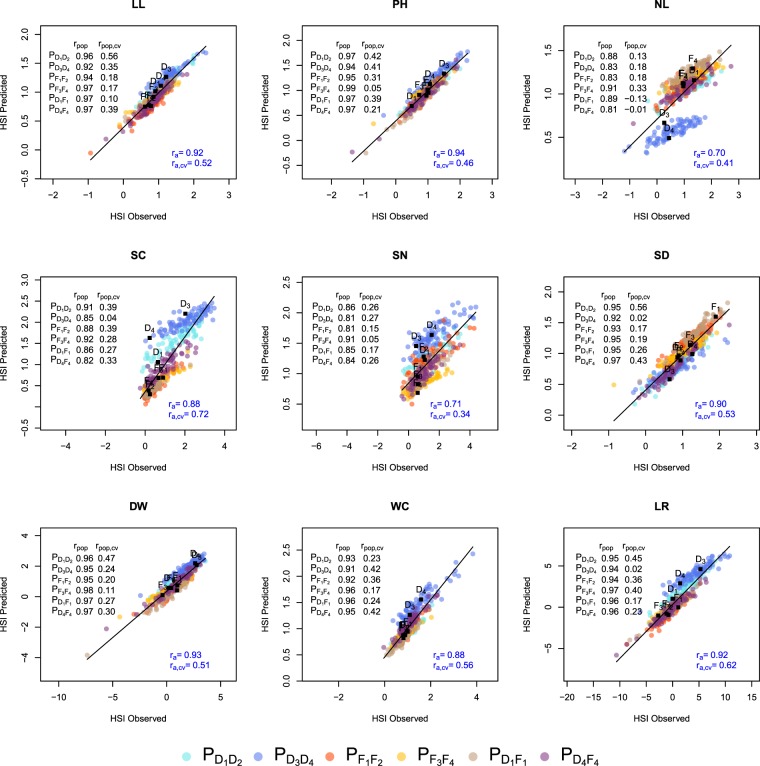
Observed versus genome-wide predicted heat susceptibility index (HSI) for each trait using RAD_482-GP:0.98_. The prediction across all populations was based on the additive model M_*A*_ applied across populations without (*r*_*a*_) or with cross validation (${r}_{a,cv}$). For the prediction within-populations, the model was built across populations but the prediction was performed within each population without (${r}_{pop}$) and with (${r}_{pop,cv}$) cross validation.

For the HSI of all traits, prediction abilities *r*_*a*_ were equal or lower for KASP_482_ than for KASP_607_ (Table 3). *r*_*a*_ calculated based on RAD_482_ were consistently higher than those for KASP_482_, independent of the GP cutoff value which was used to filter the genotype matrices. Despite this mean difference in the prediction abilities *r*_*a*_ of the different data sets and genetic models, the trend across the nine traits was the same. The prediction abilities *r*_*a*,*cv*_ varied for KASP_607_ between 0.31 (SN) and 0.70 (SC), whereas they ranged from 0.34 (SN) to 0.72 (SC) for RAD_482-GP:0.98_ (Fig. 3 and Supplementary Fig. 6). We observed a higher *r*_*a*,*cv*_ for RAD_482-GP:0.98_ than for the two other GP cutoff values (data not shown) and, therefore, used the former data set for all further analyses. The correlation between Q_ST_ and *r*_*a*_ calculated for KASP_482_ was with 0.82 significantly different from 0 ($P=0.0068$). In contrast, the correlation between Q_ST_ and *r*_*a*_ calculated based on RAD_482-GP:0.98_ was 0.19 and not significant ($P=0.62$).

Based on KASP_482_, the prediction ability across populations *r*_*a*_ was, apart from some exceptions, higher than the prediction ability calculated for each population *r*_*pop*_ (Supplementary Fig. 6). The opposite trend was observed for RAD_482-GP:0.98_ (Fig. 3). For both molecular marker types, the prediction ability *r*_*a*,*cv*_ of the M_*A*_ and M_*AD*_ genetic model did not differ significantly. However, the within-population prediction ability ${r}_{pop,BS{P}_{w}}$ of the individual population*trait combinations, was either similar or higher for the M_*A*_ compared to the M_*AD*_ model (Supplementary Figs 7–12). Therefore, we focused in this study on the former model.

**Figure 4:**
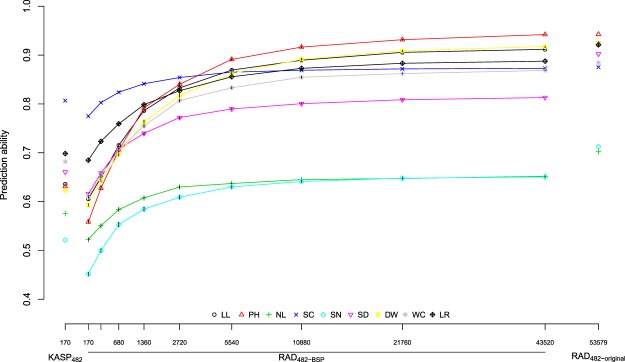
Prediction abilities (*r*_*a*_) for different numbers of RAD SNPs as well as for the entire KASP_482_ (left) and RAD_482-GP:0.98_ (right) for HSI of nine phenotypic traits. Different numbers of RAD SNPs set sizes were simulated using a resampling procedure. The black vertical bars at each points indicate the standard deviation of the prediction abilities over the 100 replications. The analyses are based on the M_*A*_ model.

An increase in the prediction ability with an increasing number of molecular markers was observed (Fig. 4). The number of markers for which the prediction ability reached a plateau differed between the examined traits.

**Figure 5:**
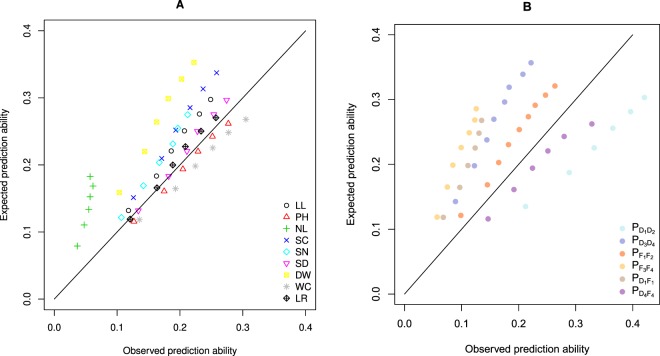
Observed vs. expected within-population prediction abilities averaged across populations (**A**) and averaged across the HSI of the different traits (**B**). The analyses are based on RAD_482-GP:0.98_ and the M_*A*_ model.

**Figure 6:**
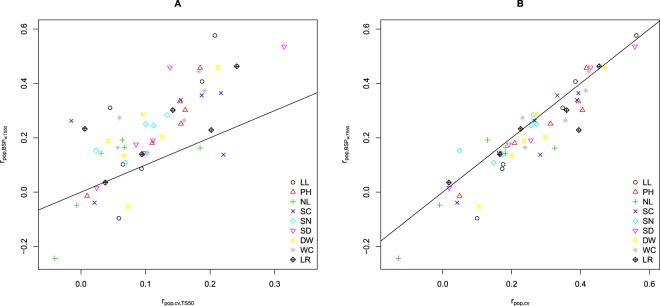
Comparison of the prediction abilities of within-population calibration (${r}_{pop,BS{P}_{w},TS50}$) with a training set (TS) of 50 genotypes with the prediction abilities based on an across-population calibration (${{\rm{r}}}_{pop,cv}$) with a TS size of 50 (**A**) and 385 (**B**) genotypes. The analyses are based on RAD_482-GP:0.98_ and the M_*A*_ model.

An increase of the within-prediction ability with an increasing size of the TS was observed for most population*trait*genetic model*molecular marker type combinations (Supplementary Figs 7–12). With a few exceptions, which showed a linear increase, the detected increase followed a logarithmic trend line (Supplementary Figs 13–15). We observed within-population prediction abilities for three traits (WC, PH and SD) that were similar to or higher than the expected abilities (Fig. 5). This was not true for the other traits. Especially, NL was predicted worse than expected. Furthermore, two populations ${{\rm{P}}}_{{D}_{1}{D}_{2}}$ and ${{\rm{P}}}_{{D}_{4}{F}_{4}}$ were predicted better than expected, whereas the other four were predicted worse than expected. The within-population prediction abilities ${r}_{pop,BS{P}_{w}}$ were for 80% of the population*trait combinations higher than those found based on a model built across the six populations (${r}_{pop,cv}$) when considering the same TS size of 50 (Fig. 6A). However, this was only true for 25% of the combinations when considering the original TS size of 385 genotypes (Fig. 6B). Across all traits and populations, the between-population predictions in the TS2_c_ scenario (TS built from two combined populations) resulted in higher prediction abilities than those of TS2_s_ (TS built from one population; Fig. 1, left). Furthermore, we observed that TS3 and especially the TS3_sr_ scenario resulted most often in the highest prediction abilities for FxF or DxD VS (Fig. 1, left). For DxF populations as VS, TS1 resulted in the highest prediction ability (Fig. 1, right).

## Discussion

For most traits, we observed a higher broad sense heritability (${{\rm{h}}}_{{\rm{a}}}^{{\rm{2}}}$) at heat compared to standard condition. This observation was due to an increased genotypic variance at heat condition while the error variance was not notably increased (data not shown). Our findings are in contrast with field-based studies in which the heritability is mostly lower at heat compared to standard condition due to an increased error variance (e.g. Cairns *et al*.^62^). This difference might be explained by field environmental factors which are more important under heat conditions, e.g. soil heterogeneity. However, these factors have low relevance under controlled conditions, e.g. in growth chambers used in our study.

The ${{\rm{h}}}_{{\rm{a}}}^{{\rm{2}}}$ values for the assessed traits were medium to high for both environmental conditions (Table 1). ${{\rm{h}}}_{{\rm{a}}}^{{\rm{2}}}$ was comparable with heritability estimates observed by Frey *et al*.^4^ and Naveed *et al*.^63^ under heat stress, and by Cerrudo *et al*.^17^ under drought stress. This observation suggests that an adequate estimation of the AEM for each genotype was achieved, which is the prerequisite for a high power to detect QTL, for genome-wide prediction, as well as to interpret differences between heterotic pools.

We observed significant ($P < 0.05$) differences in the HSI between populations derived from DxD crosses compared to populations derived from FxF crosses (Supplementary Fig. 2). Except for SD, the HSI of FxF populations were lower than that of DxD populations. This suggests that FxF populations are more tolerant to heat stress than DxD populations at seedling stage. The findings of Hallauer *et al*.^64^ suggested that the Flint pool contributed an improved chilling tolerance to cultivars bred for temperate Europe. Moreover, the results from Strigens *et al*.^65^ suggested an improved morphological and physiological adaptation of the Flint pool to chilling temperatures compared to the Dent pool. These observations along with ours suggest that genotypes of the Flint pool have a higher tolerance to temperature stress during seedling stage than genotypes of the Dent pool. This conclusion is in accordance with the results of Reimer *et al*.^9^ who observed a higher tolerance to temperature extremes during seedling stage of genotypes of the Flint pool compared to genotypes of the Dent pool.

The correlation between heat tolerance at seedling and adult stages, which was examined in a companion study^4^, was low (Supplementary Fig. 4). This finding was expected as plant performance in young stages might have limited implications on plant performance after transition from the vegetative to the generative phase. Similar trends were observed for salinity tolerance in wheatgrass^66^ or tolerance to defoliation intensity in maize^67^. Additionally, Gibert *et al*.^68^ observed that for some traits, the correlation between trait and growth changes with plant size and physiological stage. In summary, these results indicate that plant growth strategies should not be considered as constant over the entire life but is stage-dependent.

The negative correlation between the heat tolerance of maize at seedling and adult stages was also manifested on the level of heterotic pools. Genotypes derived from DxD crosses had a lower heat tolerance at seedling stage but a high heat tolerance at adult stage compared to genotypes derived from FxF crosses^4^. Therefore, both heterotic pools should be considered when increasing heat tolerance across developmental stages.

Segregating populations derived from DxF crosses are only rarely created in commercial maize breeding programs. Nevertheless, because of their different behaviour regarding heat tolerance, such populations were included in our study to test their suitability for QTL mapping and GWP. The confidence intervals of the QTL for heat tolerance at seedling stage detected in our study (Table 2) overlapped not only with QTL confidence intervals for the same trait at adult stage^4^ (Fig. 2) but also with QTL regions associated with other abiotic stresses. The confidence intervals of QTL for HSI_LL_ on chromosome 2 overlapped with a QTL associated with cold tolerance, which was identified in a metaQTL-analysis^69^. Furthermore, a QTL identified for the shoot and root dry weight and the leaf area under drought stress conditions^70^ as well as a QTL for the leaf chlorophyll content at drought stress identified by Messmer *et al*.^71^ mapped to the same genomic region on top of chromosome 2. These findings might suggest that different abiotic stresses might have similar genetic regulation mechanisms^7^. Furthermore, four out of five heat tolerance genes detected by Frey *et al*.^7^, in an RNAseq experiment, were located within the QTL confidence intervals on chromosome 2 (Supplementary Table 3, Frey *et al*.^72^). This over-representation of heat tolerance genes in a particular region and their collocation with several QTL for heat tolerance illustrates the importance of genetic mechanisms for heat tolerance available on this chromosome.

Phenotypic evaluation of genetic material for heat tolerance is difficult due to irregular and uncontrolled appearance of heat stress conditions in field experiments and is technically demanding when performed in plant growth chambers. Marker-assisted selection (MAS) using QTL for traits related to heat tolerance could supplement phenotypic selection. However, each QTL detected in our study explained with <10% a relatively small part of the phenotypic variance of the respective trait. It suggests that the heat tolerance at seedling stage is inherited as a true quantitative trait. This in turn makes the use of MAS for heat tolerance inefficient. Therefore, in a subsequent step, we evaluated the utility of GWP to improve heat tolerance of temperate maize.

With the GWP approach, we were able to explain an approximately threefold higher proportion of the genetic variance of the HSI of each trait compared to the detected QTL (Table 3). This is in accordance with earlier reports on other traits in maize^73–75^ and in other crops^24^, and illustrates the superiority of GWP over MAS for truly quantitative traits.

To quantify the potential advantage of GWP over phenotypic selection, the former can be seen as an indirect selection compared to direct phenotypic selection. The relative merit of indirect vs. direct selection can be calculated as the indirect selection response divided by the direct selection response^76^. This ratio can be rearranged as the ratio ${r}_{a,cv}/{h}_{HSI}^{2}$. GWP is superior to phenotypic selection if this ratio is >1. Assuming the same selection intensity for GWP and phenotypic selection and considering the prediction abilities (${r}_{a,cv}$ with RAD_482-GP:0.98_ and M_*A*_) of our study, the maximum relative cycle can be calculated for which identical selection gains are realised with indirect and direct selection^76,77^. The maximum relative cycle lengths for GWP were 92% (HSI_LL_), 88% (HSI_PH_), 100% (HSI_NL_), 96% (HSI_SC_), 68% (HSI_SN_), 82% (HSI_SD_), 76% (HSI_DW_), 100% (HSI_WC_), and 114% (HSI_LR_) of those of phenotypic selection. Therefore, GWP for heat stress tolerance is already equivalent or superior to phenotypic selection for three traits even without considering potential advantages due to higher selection intensities, which is very promising in light of earlier studies^27,78^. In the following, various factors influencing the prediction ability are discussed.

We found a good agreement between observed and expected prediction abilities across all studied traits (Fig. 5). However, some traits were systematically over- (NL, DW) or under-estimated (WC). One of the potential reasons for a difference between observed and expected predicted ability could be the low heritability of the traits. This was especially true for the HSI of NL which had the lowest heritability (Table 1), and which prediction ability deviated substantially from the expected values (Fig. 5). Another reason for the systematic over- and underestimations could be deviations from the highly polygenic genetic architecture of the traits which is assumed in the applied deterministic formula^58^. For such traits, one would expect that the detected QTL explained the highest proportion of the phenotypic variance. However, it is striking that the traits for which the most considerable difference between observed and expected prediction ability was found were the traits for which no QTL were detected in our study. This warrants further research.

The superiority of GWP over phenotypic selection was evaluated as described previously based on an additive genetic model. However, the F_3:4_ populations used in our study had an expected heterozygosity of 25%. In this case, it can be beneficial to investigate genetic models covering not only additive but also dominance effects when performing GWP. No obvious trend was observed regarding the superiority of one of the genetic models for the across-population prediction strategies ${r}_{a}$, ${r}_{pop}$, ${r}_{a,cv}$, and ${r}_{pop,cv}$ (data not shown). However, the ${r}_{pop,BS{P}_{w}}$ predictions abilities based on the M_*A*_ model were at least equal and, for some population*trait combinations, higher than that observed for the M_*AD*_ model (Supplementary Figs 7–12). This suggests that dominance effects were either not relevant for the studied traits or were not captured by our GBLUP model. This result was in accordance with observations made in the animal breeding field that generally, the increase of the accuracy of additive breeding values by including dominance effects was scarce^79^. Therefore, we considered only the M_*A*_ genetic model for all further analyses.

Other factors that potentially influence the prediction ability are the number and type of molecular markers that are used to to characterise the genetic material. From the three GP cutoff values examined for the RAD SNPs, RAD_482-GP:0.98_ resulted in the highest ${r}_{a,cv}$ values for most of the traits (data not shown). Our finding suggests that the mean imputation of genotypes calls with low GP is less error-prone than the genotype calls obtained by Beagle. Therefore, we chose the GP cutoff value of 0.98 for all further analyses with the RAD SNPs.

The prediction abilities ${r}_{a}$ and ${r}_{pop}$ calculated for RAD_482_ were higher than those obtained for KASP_482_, independent of the considered GP cutoff value (Table 3, Fig. 3, and Supplementary Fig. 6). Our observation is in accordance with the results of Elbasyoni *et al*.^25^ in wheat and could be due to the considerably higher number of markers that were available in the RAD data set compared to the KASP data set. This was confirmed by the increasing prediction abilities observed for increasing numbers of RAD SNPs (Fig. 4). A high number of markers increases the precision of the **K** estimates, increases LD between markers and QTL, as well as ensures a better genome representation.

A second observation leading to the same conclusion was that Q_ST_ was significantly correlated with *r*_*a*_ calculated for KASP_482_ but not with *r*_*a*_ calculated for RAD_482-GP:0.98_. This finding suggests that the prediction abilities obtained with the KASP SNPs are predominantly due to the modeling of genetic relationships and are therefore higher when the ratio of genetic variance among- vs. within-populations increases. In contrast, the prediction abilities obtained with RAD SNPs are to a higher extent due to LD between marker and QTL alleles and to a lower extent due to the modeling of genetic relationship and thus do not correlate with Q_ST_.

Finally, the prediction abilities *r*_*a*_ calculated for KASP_482_ were with 0.72 also significantly (P = 0.028) correlated with ${h}_{HSI}^{2}$, which is in accordance with the results of Poland *et al*.^80^, Hayes *et al*.^81^, and De Moraes *et al*.^26^. However, the prediction ability *r*_*a*_ calculated for RAD_482-GP:0.98_ were not significantly (P = 0.14) correlated with ${h}_{HSI}^{2}$. This agrees theoretical considerations^82^ and empirical studies^83^ and can be explained by an increased proportion of the prediction abilities caused by LD between markers and QTL when a high number of molecular markers was used. Therefore, all discussions hereafter are based on RAD_482-GP:0.98_.

Further factors that have a high impact on the prediction abilities are the size and composition of the training set and, thus, were examined in our study. For most trait*population combinations, we observed an increase of the prediction ability ${r}_{pop,BS{P}_{w}}$ with an increasing size of the training set (Supplementary Figs 7–13). This is in accordance with the results of Van Raden *et al*.^84^ and Technow *et al*.^27^. However, in contrast to these authors, we did not observe a plateau with an increasing size of the TS. One reason for this could be that our training set size is small and the plateau was not yet reached. Nervertheless, the TS size of this study corresponds to that typically used in commercial maize breeding programs.

We observed significant ($P < 0.05$) differences in the heat tolerance between the two heterotic pools. Therefore, we were interested in examining the potential of prediction between populations (inter-population calibration) and heterotic pools (inter-pool calibration) in comparison with the previously discussed within-population calibration. The latter resulted across all population*trait combinations in higher prediction abilities ${r}_{pop,BS{P}_{w}}$ than the inter-population calibration, independent of the TS used for the ${r}_{pop,BS{P}_{b}}$. This is in accordance with the results of Technow *et al*.^27^. The most likely reason for this is that LD patterns are not consistent between heterotic pools^85,86^. To identify markers that are in LD with QTL across different heterotic pools, an even higher number of markers might be required^86,87^. Nevertheless, as situations in which within-population prediction rarely appear in commercial breeding programs, we evaluated various scenarios for ${r}_{pop,BS{P}_{b}}$.

We observed a considerably lower prediction ability for intra-pool calibration (${r}_{pop,BS{P}_{b}}$ based on TS1; Fig. 1, left) than for the intra-population calibration (${r}_{pop,BS{P}_{w}}$). This was more pronounced than expected according to Habier *et al*.^54^. Our observation can be due to the fact that the parental inbreds used to develop segregating populations in our study were selected such that they show a high variation for heat tolerance related traits not only across all eight inbreds but also across the inbreds of one heterotic pool. Therefore, a high variation between the populations was observed even when the parental inbreds were from the same heterotic pool.

Compared to TS1, we observed a minor increase of the prediction ability when using an inter-pool calibration (TS2; Fig. 1, left). This was especially true when the TS was composed of two populations (TS2_c_). Moreover, this finding might be explained by the fact that the four Flint and Dent parental inbred lines used to develop the segregating populations were chosen to be as diverse as possible.

Compared to the results of TS2 (_s_ and _c_), the prediction abilities for the mixed-pool calibration (TS3) were even higher. A part of this increase was due to the fact that the DxF populations shared parental inbreds with the DxD and FxF populations. Our observation that the prediction ability for a scenario with shared parental inbred (TS3_sr_) were higher than for a scenario with no shared parental inbreds between TS and VS (TS3_su_; Fig. 1, left) supported this explanation. In addition to the fact that TS and VS have one common parental inbred, the higher prediction ability for TS3 compared to TS2 (_s_ and _c_) might be due to the higher segregation variance of the mixed populations (Supplementary Table 2 and Supplementary Fig. 5).

This aspect was studied more in detail by examining the prediction ability for mixed populations (DxF) using different types of TS (Fig. 1, right). The TS with combined FxF and DxD populations which were partially derived from common parents with the VS (TS3) performed better than combined FxF and DxD populations that had no parental inbred in common with the VS (TS2). This further emphasises parental inbreds shared between the populations of the TS and VS is an important component for the success of genomic prediction. However, we observed the highest prediction ability for mixed populations when using other mixed populations as TS even if when had no common parents (TS1). One hypothesis to explain this result is the high diversity of such mixed populations (Supplementary Table 2). Our findings suggest that commercial maize breeding companies who create DxF populations to e.g. increase the per-se performance of the Flint pool with Dent material or to introgress earliness in Dent inbreds with Flint material can use such populations as TS to predict the original heterotic pools.

Generally, the critical aspect when selecting the TS in routine plant breeding programs is the limited size of the segregating populations which hampers either the building of a TS of suitable size or reduces relatedness between TS and VS if several segregating populations are used to build the TS. Therefore, we have evaluated the potential of across-pools and -populations calibrations. ${r}_{pop,BS{P}_{w}}$ was for 80% of the population*trait combinations better than a model built on the six populations (${r}_{pop,cv}$) if considering the same TS size of 50. However, this was only the case for 25% of the combinations if considering the original TS size of 385 genotypes (Fig. 6). This implies that if genomic prediction is used to select among the genotypes of one single population and if the data for the TS must be generated de novo, the use of within-population prediction is the most promising approach. Nevertheless, in many cases, a commercial maize breeding company has multiple connected segregating populations for which predictions should be obtained. For such a scenario, our results clearly indicate that a combination of populations in one TS, also across different heterotic pools, increases the prediction ability compared to the use of within-population calibration (Fig. 6).

## Conclusion

In this study, we provided a basis for future research on genome regions and candidate genes involved in heat stress tolerance of maize seedlings. Antagonistic pleiotropy between heat tolerance at seedling and adult stages was observed in genomic hot-spot regions, especially on chromosome 2. Although six QTL were detected in our study, these explained a very small proportion of the phenotypic variance. Therefore, marker-assisted selection is not promising for the traits evaluated in our study. On the contrary, the prediction abilities observed for GWP encourages the use of such approaches for heat tolerance at seedling stage, especially if genotypic characterisation was carried out with a high number of markers, e.g. from RAD sequencing. Furthermore, our results suggest that the combination of populations from different heterotic pools in one training set is a promising approach to increase the prediction ability for heat tolerance traits, especially if the population in the TS share parental genotypes with those of the VS. Finally, we demonstrated that for the examined traits, segregation populations derived from inter-pool crosses are very suitable to predict populations derived from intra-pool crosses.

## Supplementary information

Supplementary Information

Dateset 1 (KASP data)

Dataset 2 (Phenotypic data)

## Data Availability

The sequencing datasets generated and analysed in the current study are available in the NCBI Sequence Read Archive (SRA) repository, https://www.ncbi.nlm.nih.gov/sra/?term=PRJNA564701. All phenotyping and genotyping data generated or analysed during this study are included as supplementary information files.
