# Supplementary material for: QTL mapping and genome-wide prediction of heat tolerance in multiple connected populations of temperate maize
Source: Sci Rep. 2019 Oct 8;9:14418. doi: 10.1038/s41598-019-50853-2 (PMC6783442; doi:10.1038/s41598-019-50853-2)
Supplement: Supplementary file 1 — Supplementary Information [file 41598_2019_50853_MOESM1_ESM.pdf]

SUPPLEMENTARY INFORMATION: QTL mapping and genome-wide prediction of heat tolerance in multiple connected populations of temperate maize

Delphine Van Inghelandt[+,1], Felix P. Frey[+,2,3], David Ries[1], Benjamin Stich[1,2,*]

September 26, 2019

[1]Institute for Quantitative Genetics and Genomics of Plants, Cluster of Excellence on Plant Sciences (CEPLAS), Heinrich Heine University, 40225 Düsseldorf, Germany;
[2]Max Planck Institute for Plant Breeding Research, 50829 Köln, Germany;
[3]Current Address: Crop Functional Genomics, Institute of Crop Science and Resource Conservation, University of Bonn, 53113 Bonn, Germany

[+]These authors contributed equally to this work.
[*] Corresponding author: Prof. Dr. Benjamin Stich,
e-mail: benjamin.stich@hhu.de

Supplementary material SM1

Variant filtering: For the first round of filtering, GATK's SelectVariants and VariantFiltration were used. Thereby, only biallelic sites with a quality by depth (QD) value > 2.0 and a mapping quality (MQ) value > 40.0 were kept, and genotype calls covered by less than four reads were set to "not called". Since, duplicate reads should not be removed from single end RAD-Seq data, ambiguous genotype calls with a coverage of greater than 50 were removed by a coverage filter using vcftools [1] with the –maxDP parameter set to 50. Afterwards, the fraction of missing genotype calls per sample was calculated with vcftools –missing-indv and all samples with a missingness > 0.95 were removed using bcftools view [2]. Finally, vcftools was applied with the parameters –max-missing 0.20 and –maf 0.05. In the last step only variant sites with an allele call in at least 80% of the samples, and a minor allele frequency of greater then 0.05 were kept.

Supplementary Table 1: Broad sense heritability (h^2_{pop}) of the studied traits for each environmental condition and for the heat susceptibility index (HSI) calculated separately for each population.

Trait	Populations					
	$P_{D_1D_2}$	$P_{D_3D_4}$	$P_{F_1F_2}$	$P_{F_3F_4}$	$P_{D_1F_1}$	$P_{D_4F_4}$
Standard condition						
Leaf length (LL)	0.67	0.67	0.59	0.65	0.73	0.28
Plant height (PH)	0.56	0.60	0.54	0.47	0.63	0.73
Number of leaves (NL)	0.61	0.03	0.56	0.62	0.65	0.34
Leaf scorching (SC)	0.11	0.25	0.00	0.00	0.00	0.00
Leaf senescence (SN)	0.49	0.60	0.57	0.68	0.47	0.24
Leaf greenness (SD)	0.76	0.61	0.69	0.73	0.78	0.39
Shoot dry weight (DW)	0.60	0.65	0.67	0.58	0.76	0.51
Shoot water content (WC)	0.48	0.43	0.46	0.32	0.49	0.58
Leaf growth rate (LR)	0.06	0.29	0.48	0.63	0.67	0.41
Heat condition						
LL	0.74	0.75	0.78	0.71	0.68	0.05
PH	0.80	0.75	0.75	0.50	0.60	0.60
NL	0.66	0.67	0.52	0.45	0.67	0.03
SC	0.66	0.59	0.59	0.58	0.70	0.14
SN	0.49	0.76	0.61	0.34	0.46	0.33
SD	0.60	0.57	0.70	0.66	0.71	0.00
DW	0.71	0.77	0.82	0.70	0.76	0.39
WC	0.59	0.59	0.46	0.23	0.50	0.58
LR	0.59	0.64	0.64	0.22	0.40	0.21
HSI						
LL	0.40	0.61	0.48	0.48	0.46	0.48
PH	0.58	0.62	0.26	0.26	0.46	0.38
NL	0.37	0.54	0.24	0.19	0.23	0.16
SC	0.67	0.47	0.51	0.55	0.60	0.55
SN	0.36	0.55	0.62	0.51	0.34	0.31
SD	0.50	0.29	0.60	0.57	0.47	0.49
DW	0.61	0.64	0.62	0.52	0.55	0.58
WC	0.44	0.62	0.35	0.35	0.30	0.55
LR	0.55	0.42	0.34	0.48	0.50	0.35

Supplementary Table 2: Mean modified Roger's distance between populations calculated from $KASP_{482}$ (above diagonal; after \) and $RAD_{482\text{-}GP:0.98}$ (below diagonal; before \) and within populations (on the diagonal), gene diversity D, and fixation index G_{ST} of the populations and the whole set.

	$P_{D_1D_2}$	$P_{D_3D_4}$	$P_{F_1F_2}$	$P_{F_3F_4}$	$P_{D_1F_1}$	$P_{D_4F_4}$	Overall
$P_{D_1D_2}$	0.14\0.27	0.38	0.44	0.43	0.38	0.41	
$P_{D_3D_4}$	0.29	0.17\0.23	0.45	0.46	0.42	0.37	
$P_{F_1F_2}$	0.39	0.42	0.23\0.28	0.38	0.38	0.42	
$P_{F_3F_4}$	0.35	0.39	0.28	0.09\0.29	0.39	0.39	
$P_{D_1F_1}$	0.27	0.34	0.29	0.28	0.18\0.30	0.43	
$P_{D_4F_4}$	0.31	0.27	0.36	0.25	0.33	0.18\0.31	
D KASP	0.21	0.16	0.26	0.23	0.24	0.28	0.39
D RAD	0.16	0.12	0.19	0.16	0.20	0.19	0.16
G_{ST} KASP	0.69	0.63	0.74	0.71	0.74	0.79	0.58
G_{ST} RAD	0.71	0.63	0.72	0.70	0.78	0.77	0.57

Supplementary Table 3: Heat tolerance genes described by Frey *et al.* [3] that are located within QTL confidence intervals for heat susecptibility index (HSI) of five traits (Leaf length: LL, Plant height: PH, Leaf scorching: SC, Leaf greenness: SD, Leaf growth rate: LR) at seedling stage and for female flowering: FF, male flowering: MF at adult stage [4].

Gene	Chr	QTL seedling	QTL adult	Description
GRMZM2G148998	2	$Q_{HSI:LLa}$	$Q_{HSI:FF}$,$Q_{HSI:MFa}$	Uncharacterized protein
GRMZM2G430362	2	$Q_{HSI:LLa}$		ATP-dependent RNA helicase SUV3
GRMZM2G035063	2	$Q_{HSI:LR}$		Chaperonin
GRMZM2G099425	2	$Q_{HSI:LR}$		Calcium-dependent protein kinase, isoform AK1; Uncharacterized protein
GRMZM2G436710	5	$Q_{HSI:SD}$	$Q_{HSI:MFb}$	Uncharacterized protein

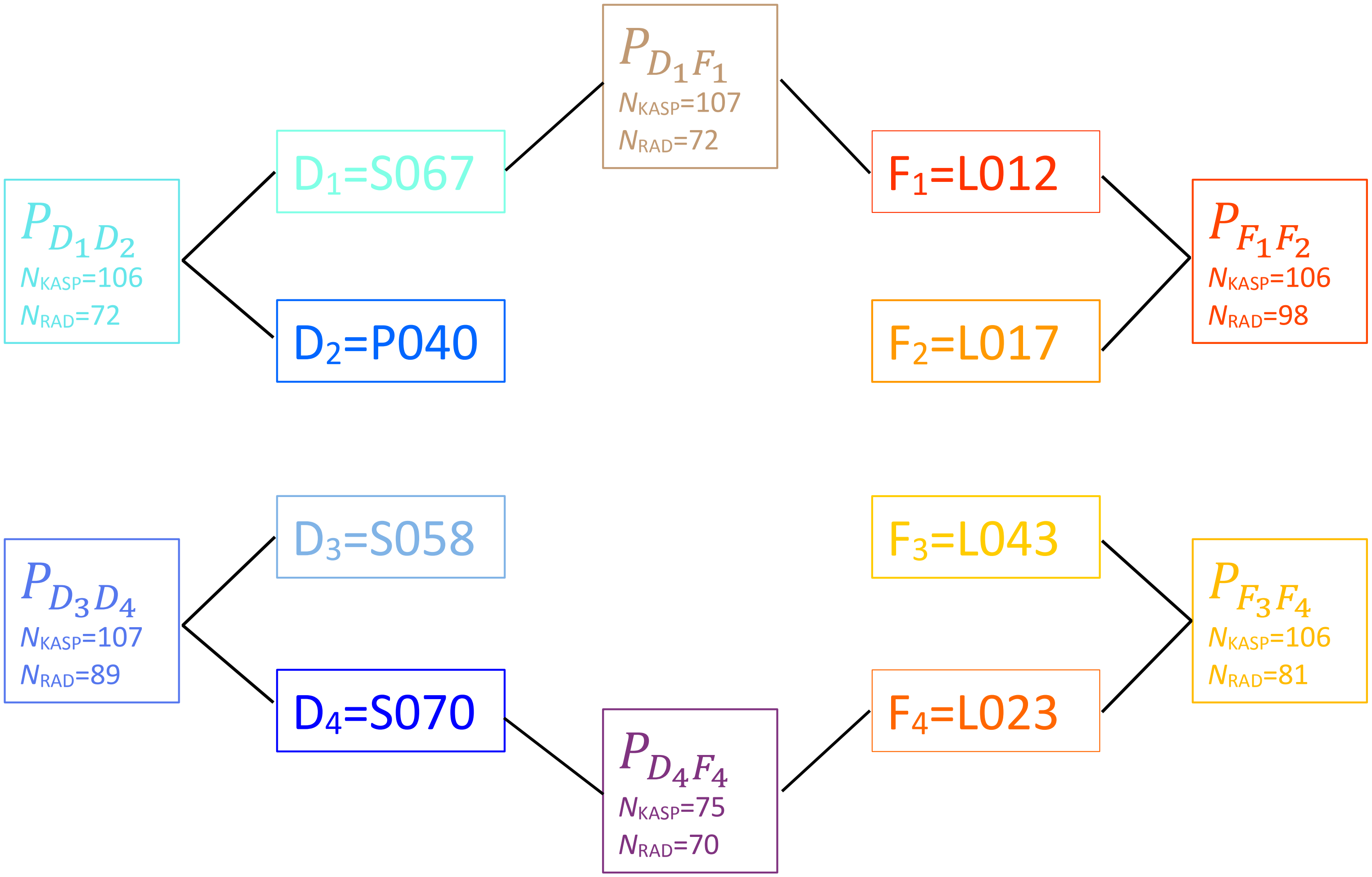

Supplementary Figure 1: Crossing scheme used to create six segregating populations derived from four Dent (S067, P040, S058 and S070, in bluish) and four Flint (L012, L017, L043 and L023, in yellowish/redish) inbred lines, where *N* is the number of progenies that were genotyped with two different types of SNP markers.

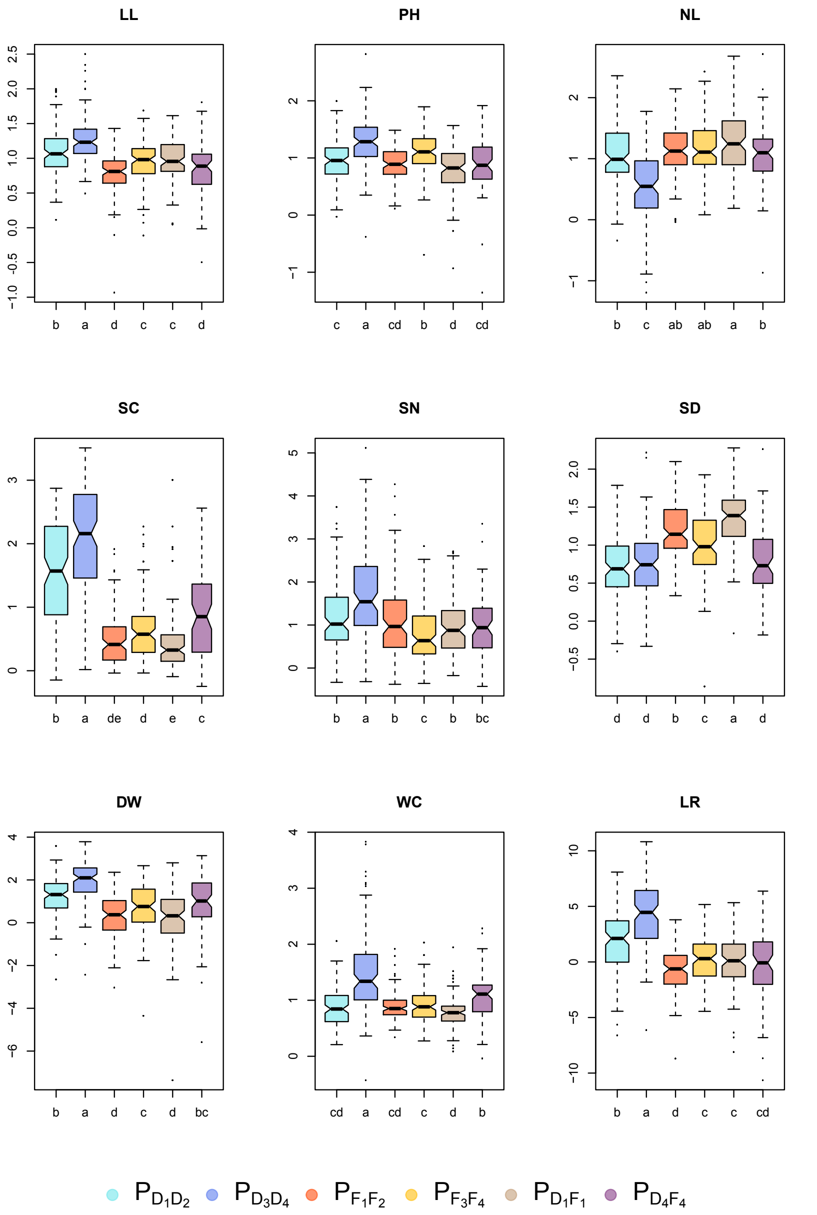

Supplementary Figure 2: Boxplot of the heat susceptibility index (HSI) of nine traits (leaf length (LL), plant height (PH), number of leaves (NL), leaf scorching (SC), leaf senescence (SN), leaf greenness (SD), shoot dry weight (DW), shoot water content (WC) and leaf growth rate (LR)) for the six segregating populations. Populations marked with different letters on the X axis have significantly ($P < 0.05$) different means.

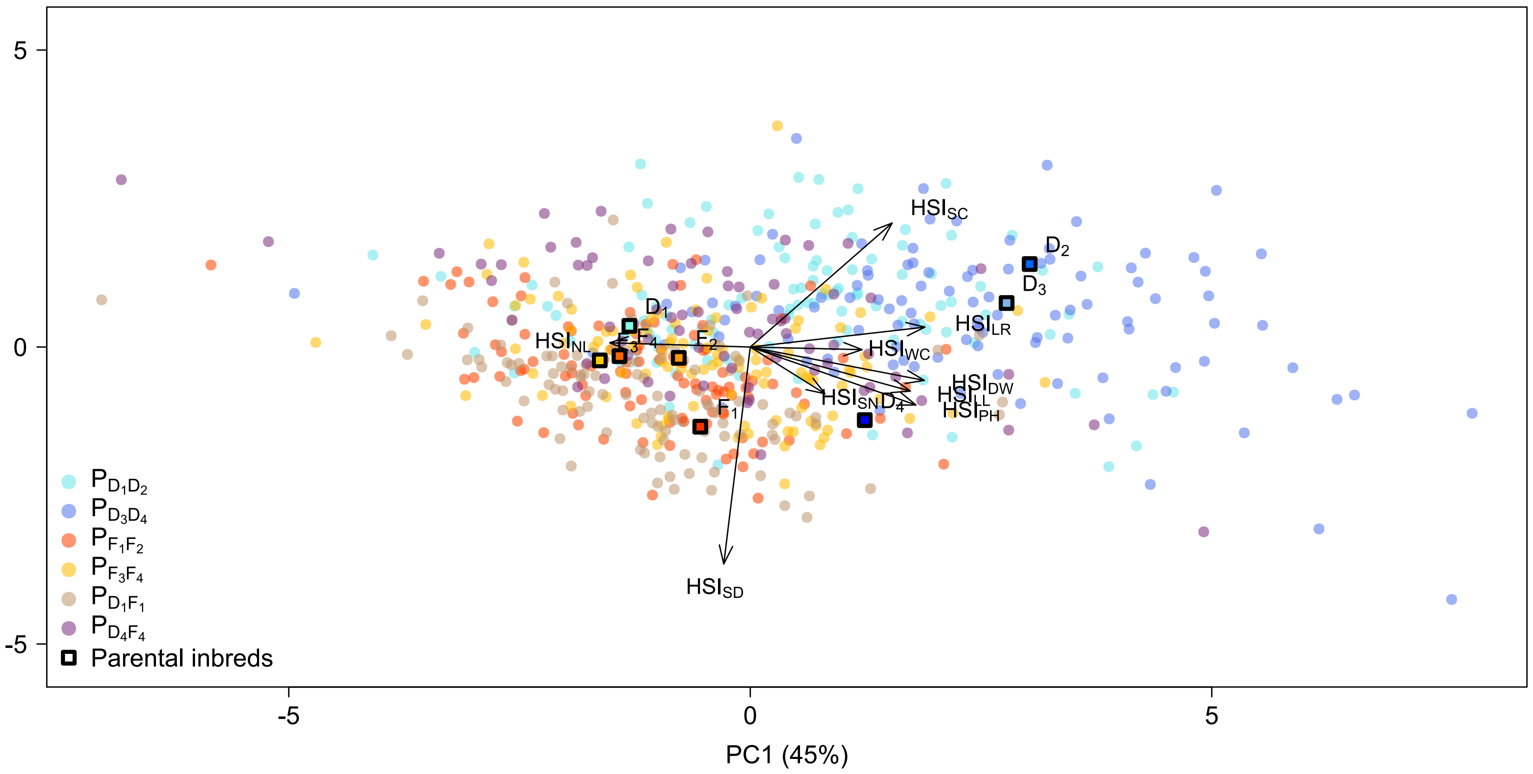

Supplementary Figure 3: Plot of the first two principal components (PC1 and PC2) of a principal component analysis performed on the heat susceptibility indexes (HSI) of the nine assessed traits: leaf length (LL), plant height (PH), number of leaves (NL), leaf scorching (SC), leaf senescence (SN), leaf greenness (SD), shoot dry weight (DW), shoot water content (WC) and leaf growth rate (LR). The numbers in parentheses refer to the proportion of variance explained by the PC.

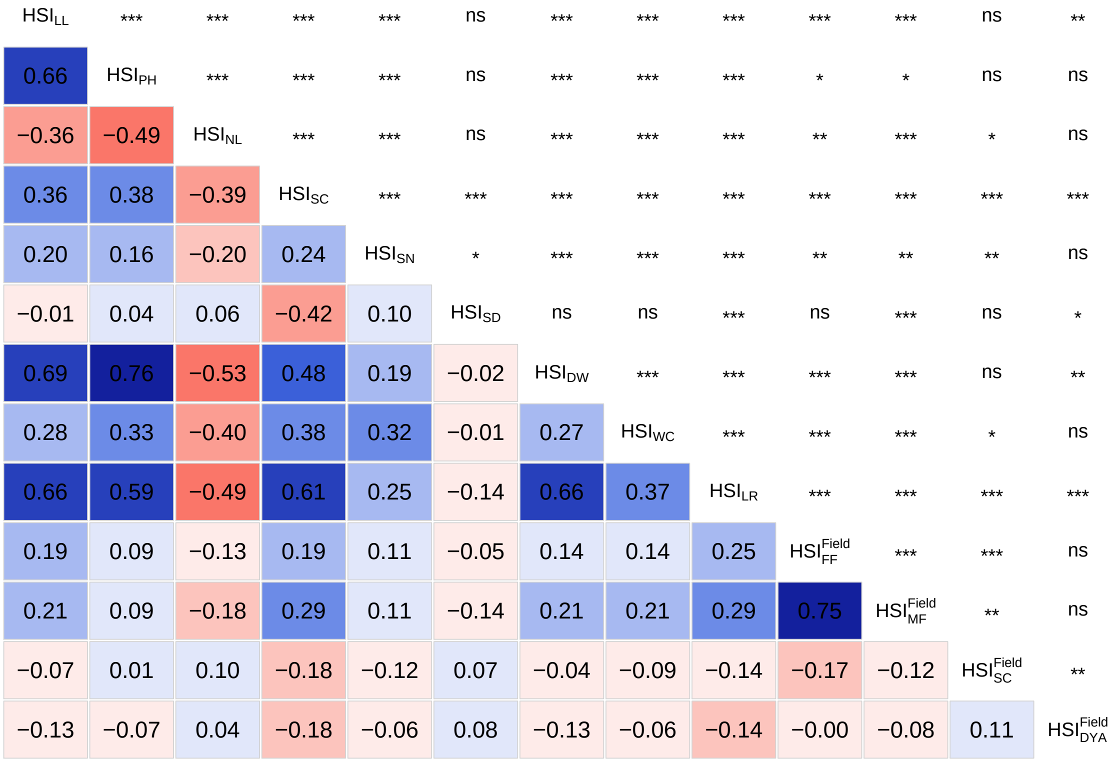

Supplementary Figure 4: Correlations between the heat susceptibility indexes (HSI) of nine seedling traits (leaf length (LL), plant height (PH), number of leaves (NL), leaf scorching (SC), leaf senescence (SN), leaf greenness (SD), shoot dry weight (DW), shoot water content (WC) and leaf growth rate (LR)), and the HSI of traits assessed during adult stage under field conditions [4] (time to female (FF) and male flowering (MF), leaf scorching (SC) and dry grain yield adjusted with the time to female flowering (DYA)) across all segregating populations. Significance ($P <= 0.05, 0.01, 0.001$) of correlations is denoted with *, **, ***, respectively or with ns for not significant.

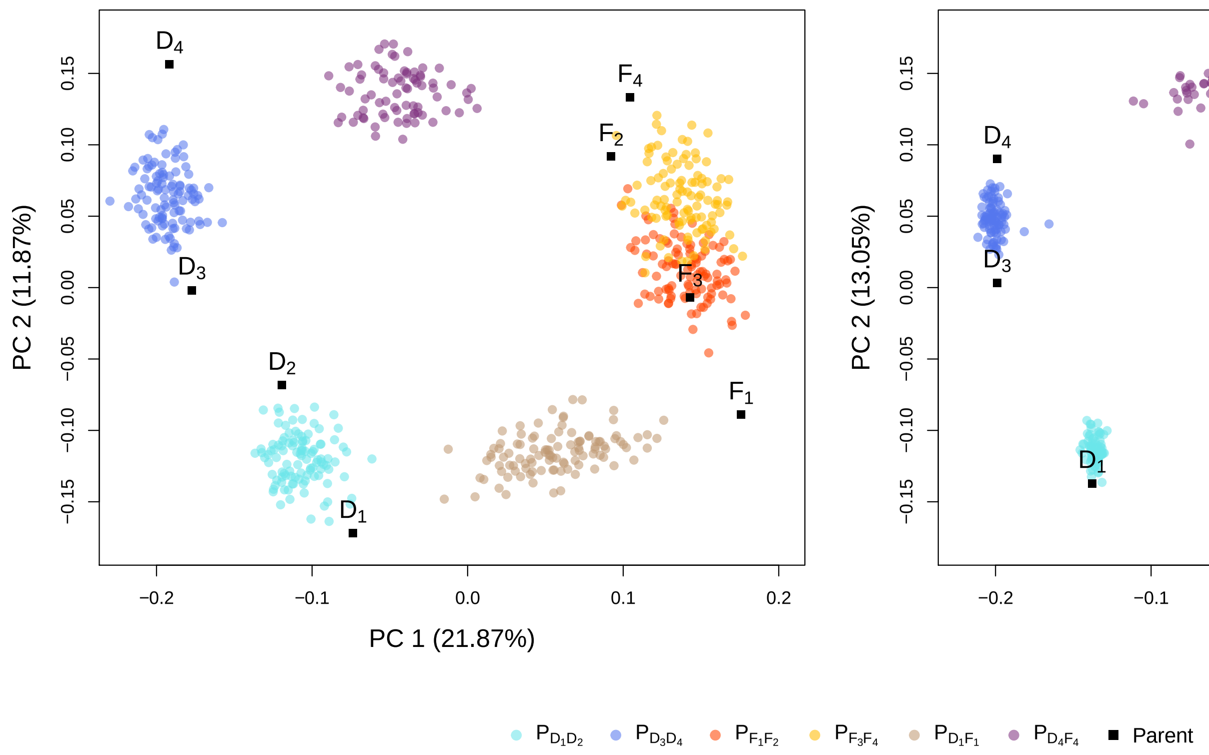

Supplementary Figure 5: Plot of the first two principal components (PC1 and PC2) of a principal component analysis of six segregating maize populations based on modified Roger's distance estimates calculated from $KASP_{607}$ (left) as well as $RAD_{482\text{-}GP:0.98}$ (right), where available parental inbreds were included as well. The numbers in parentheses refer to the proportion of variance explained by the PC.

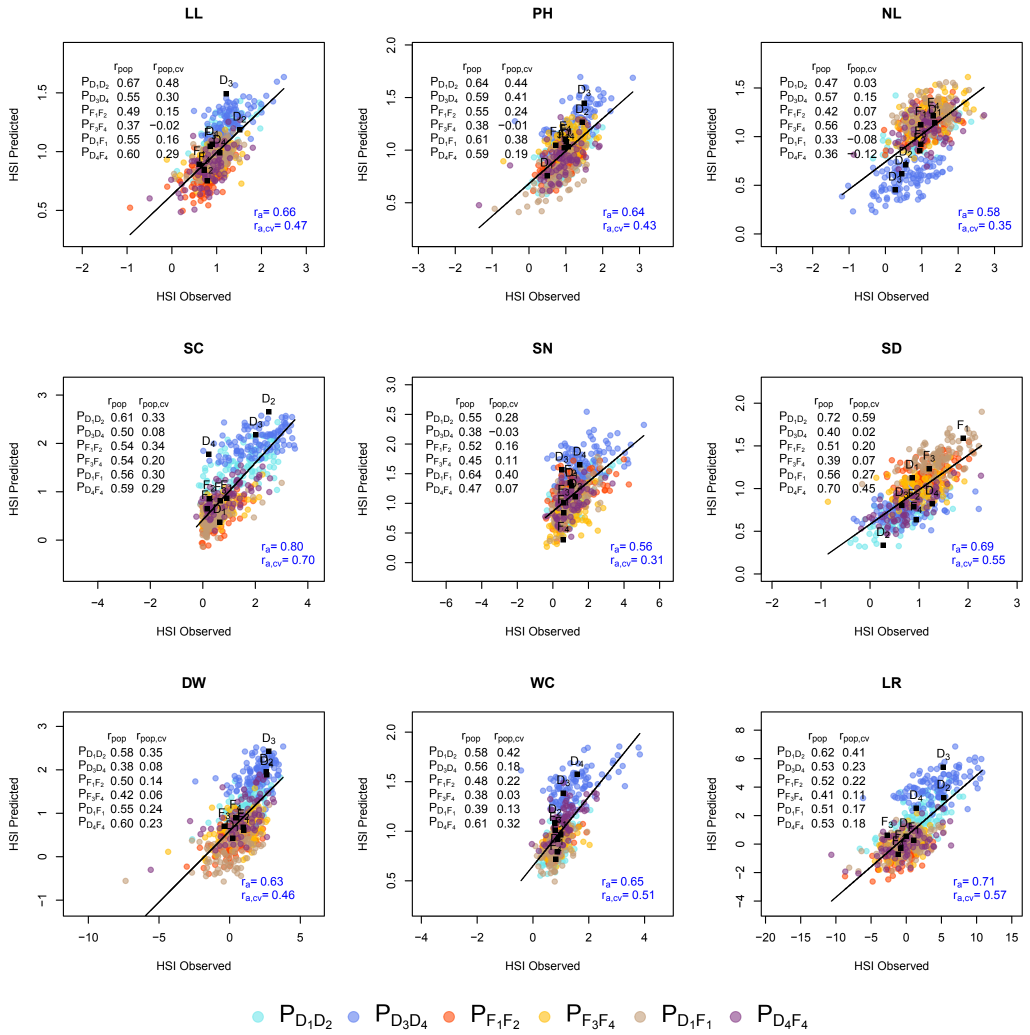

Supplementary Figure 6: Observed versus genome-wide predicted heat susceptibility index (HSI) for each trait using $KASP_{607}$. The prediction across all populations was based on the additive model M_A applied across populations without (r_a) or with cross validation ($r_{a,cv}$). For the prediction within-populations, the model was built across populations but the prediction was performed within each population without (r_{pop}) and with ($r_{pop,cv}$) cross validation.

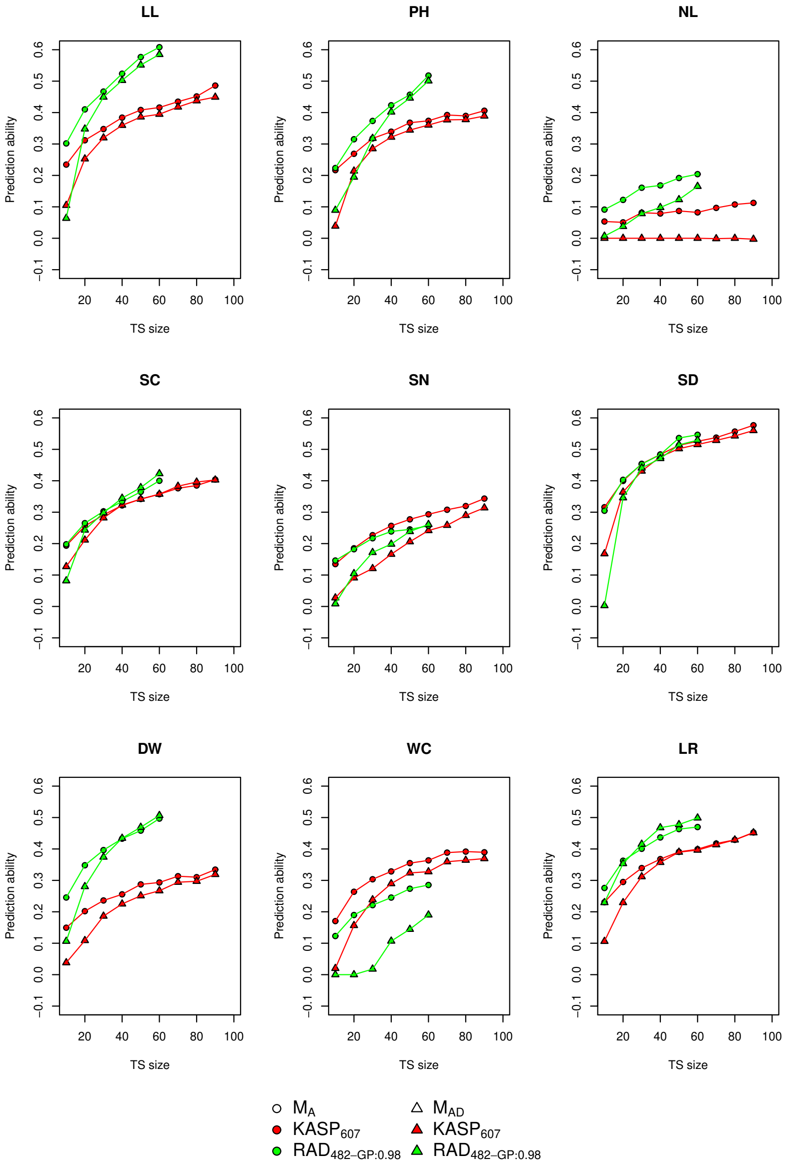

Supplementary Figure 7: Within-population prediction abilities r_{pop,BSP_w} for heat susceptibility index of nine traits within $P_{D_1D_2}$ for different training set (TS) sizes based on two different molecular marker types ($KASP_{607}$ and $RAD_{482\text{-GP:0.98}}$) and two genetic models (M_A and M_{AD}).

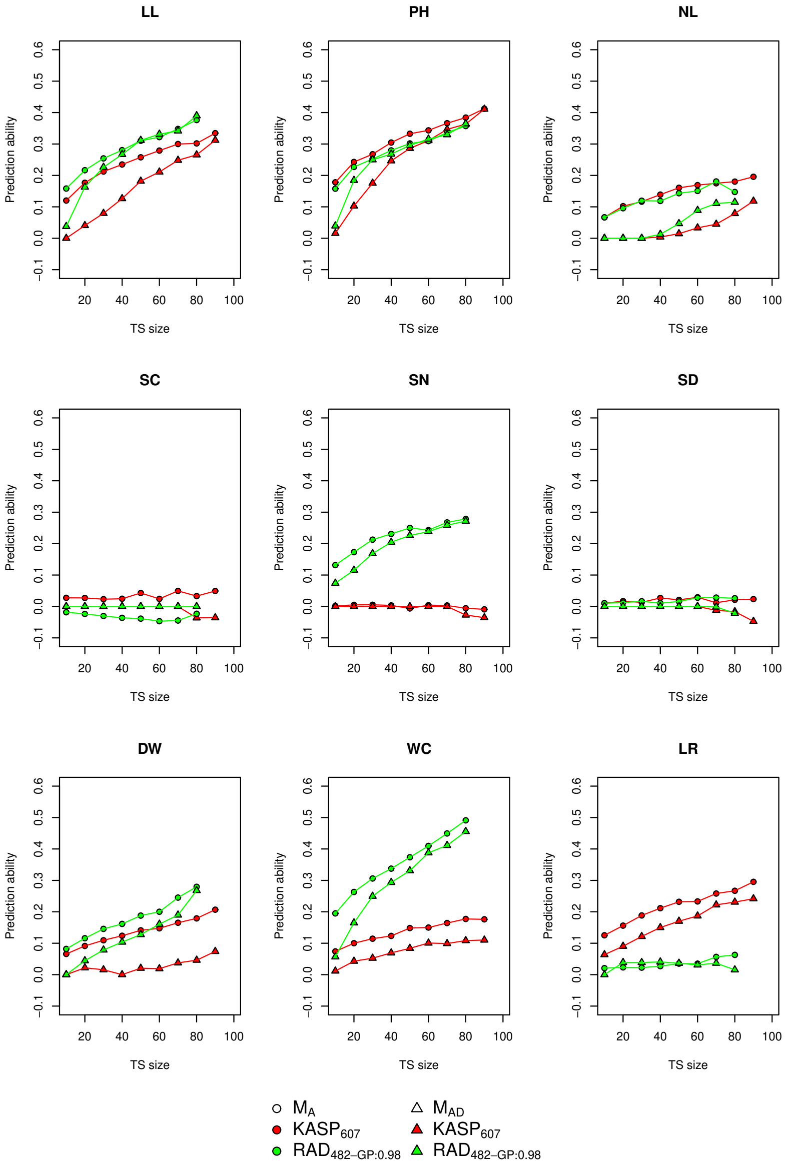

Supplementary Figure 8: Within-population prediction abilities r_{pop,BSP_w} for heat susceptibility index of nine traits within $\mathrm{P}_{D_3D_4}$ for different training set (TS) sizes based on two different molecular marker types (KASP_{607} and $\mathrm{RAD}_{482\text{-GP:0.98}}$) and two genetic models (M_A and M_{AD}).

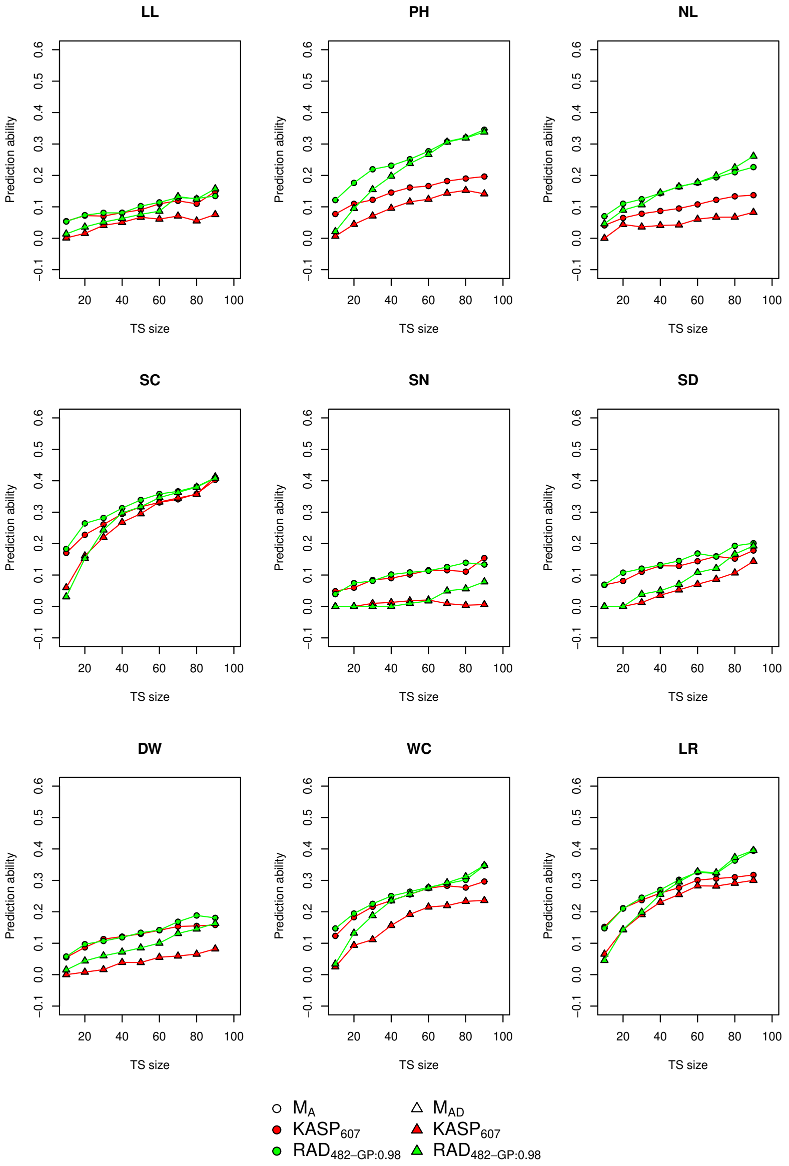

Supplementary Figure 9: Within-population prediction abilities r_{pop,BSP_w} for heat susceptibility index of nine traits within $P_{F_1F_2}$ for different training set (TS) sizes based on two different molecular marker types ($KASP_{607}$ and $RAD_{482\text{-}GP:0.98}$) and two genetic models (M_A and M_{AD}).

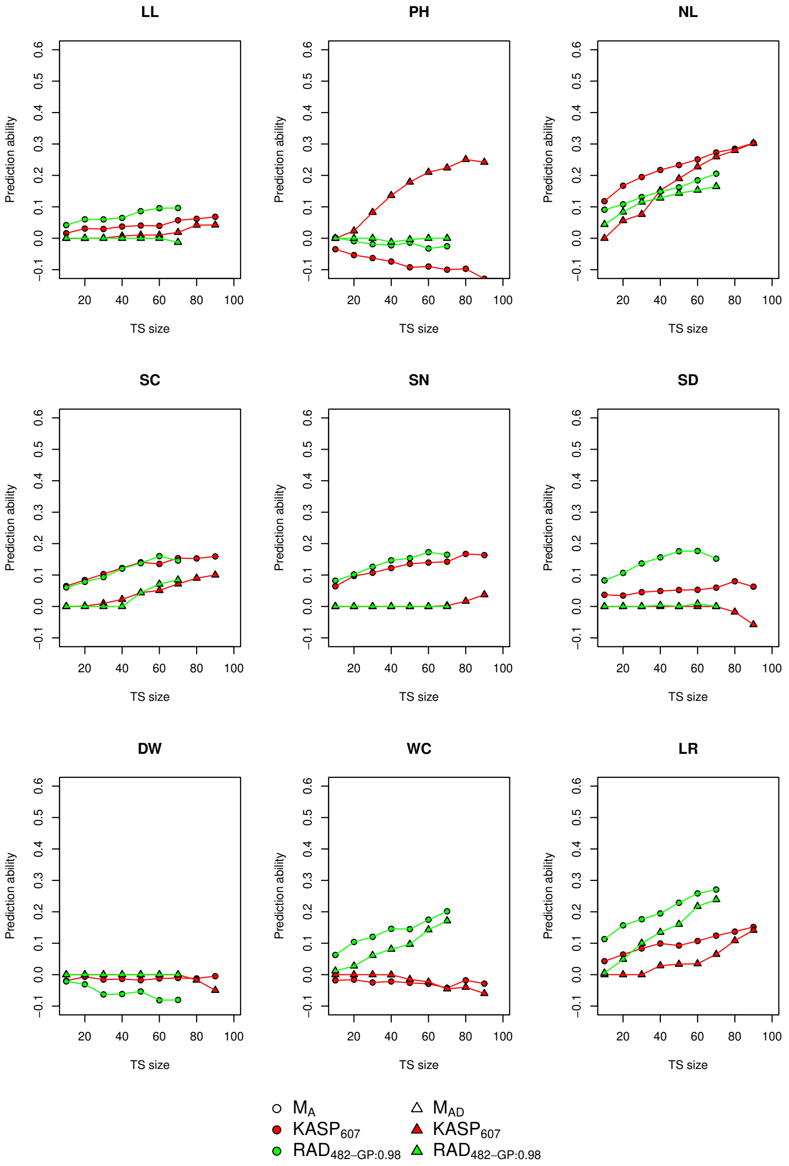

Supplementary Figure 10: Within-population prediction abilities r_{pop,BSP_w} for heat susceptibility index of nine traits within $P_{F_3F_4}$ for different training set (TS) sizes based on two different molecular marker types ($KASP_{607}$ and $RAD_{482\text{-GP:0.98}}$) and two genetic models (M_A and M_{AD}).

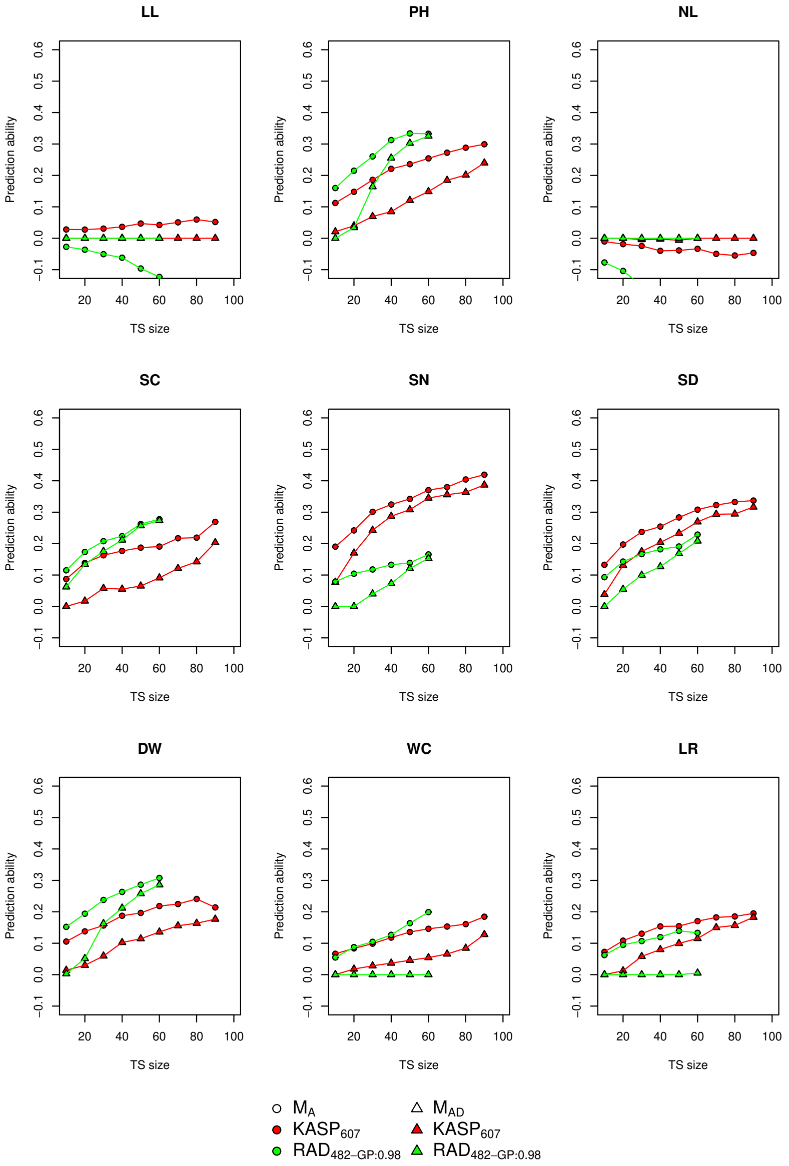

Supplementary Figure 11: Within-population prediction abilities r_{pop,BSP_w} for heat susceptibility index of nine traits within $P_{D_1F_1}$ for different training set (TS) sizes based on two different molecular marker types ($KASP_{607}$ and $RAD_{482\text{-GP:0.98}}$) and two genetic models (M_A and M_{AD}).

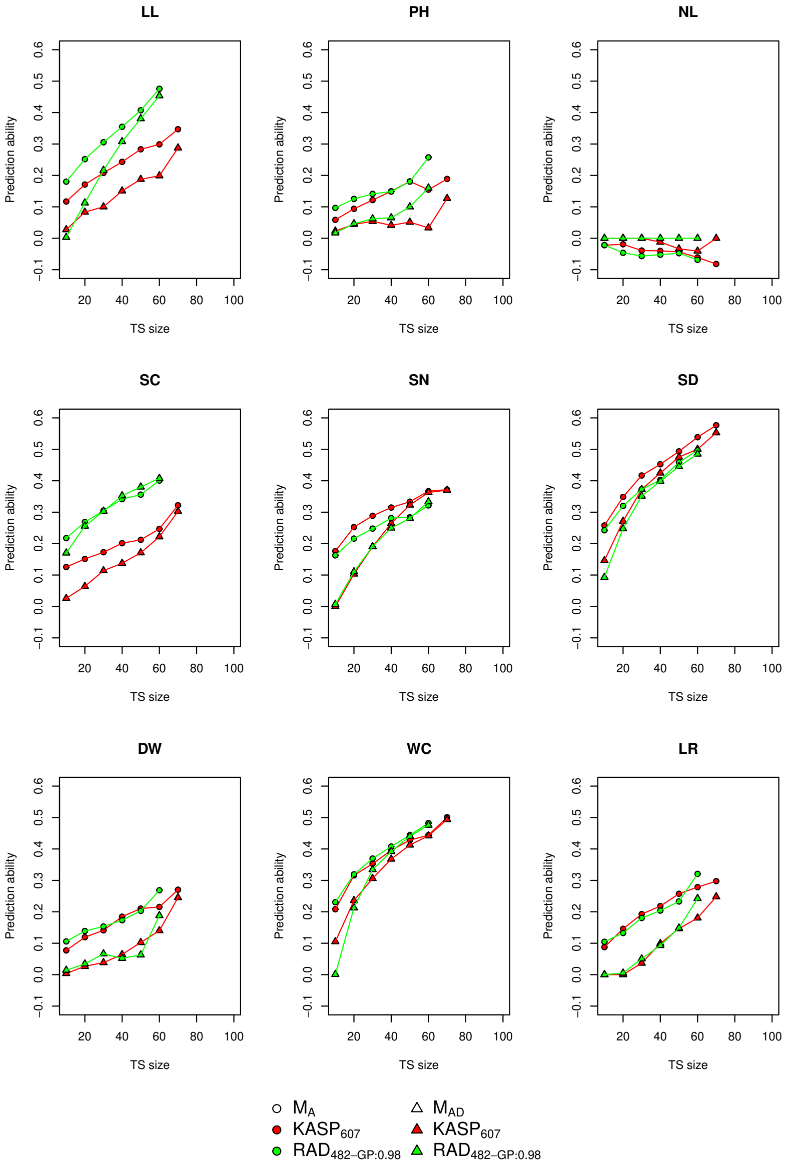

Supplementary Figure 12: Within-population prediction abilities r_{pop,BSP_w} for heat susceptibility index of nine traits within $P_{D_4F_4}$ for different training set (TS) sizes based on two different molecular marker types ($KASP_{607}$ and $RAD_{482\text{-}GP:0.98}$) and two genetic models (M_A and M_{AD}).

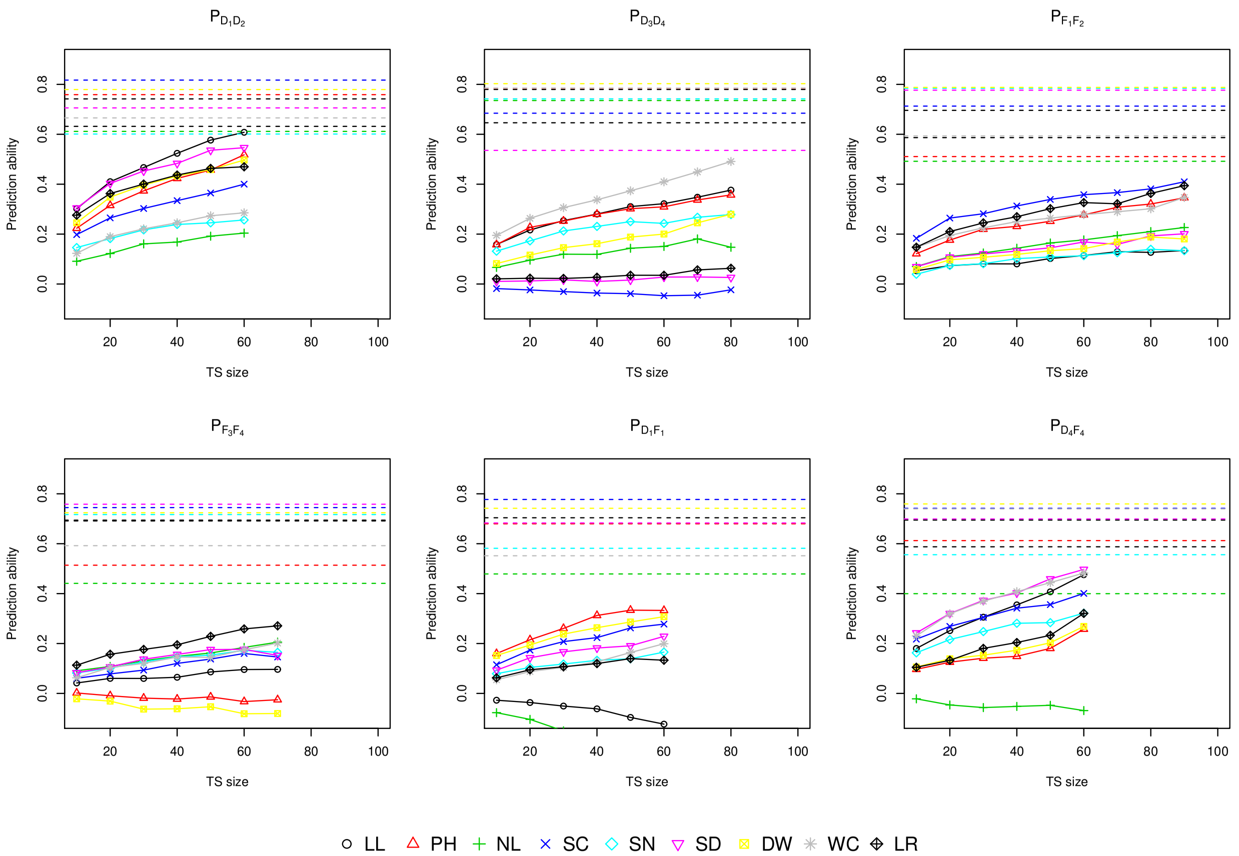

Supplementary Figure 13: Within-population prediction abilities (r_{pop,BSP_w}) using a boostrapping procedure to simulate different sizes of the training set (TS). The analyses are based on $RAD_{482-GP:098}$ and the M_A model. The dashed lines indicate phenotypic accuracies calculated as the square root of the heritability of the HSI for each trait (leaf length (LL), plant height (PH), number of leaves (NL), leaf scorching (SC), leaf senescence (SN), leaf greenness (SD), shoot dry weight (DW), shoot water content (WC) and leaf growth rate (LR)) in each population.

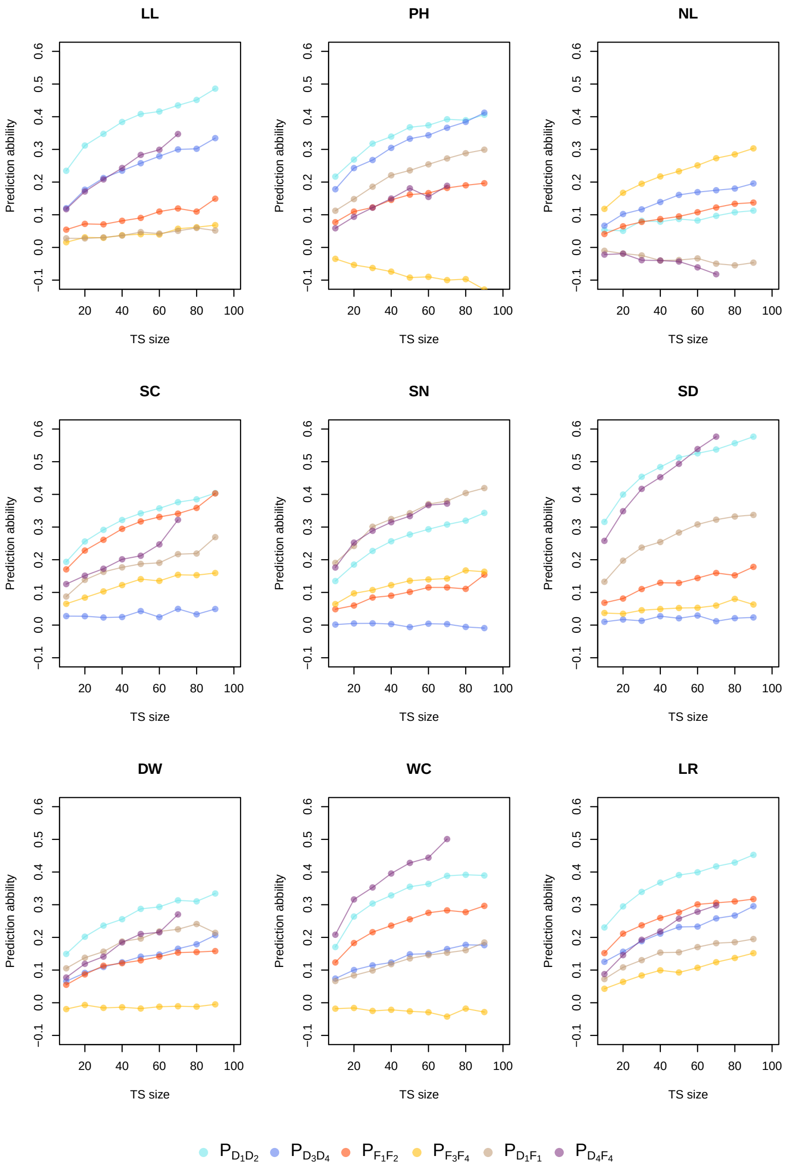

Supplementary Figure 14: Within-population prediction abilities r_{pop,BSP_w} for heat susceptibility index calculated for nine traits for different training set (TS) sizes for each population based on $KASP_{607}$ and the M_A model.

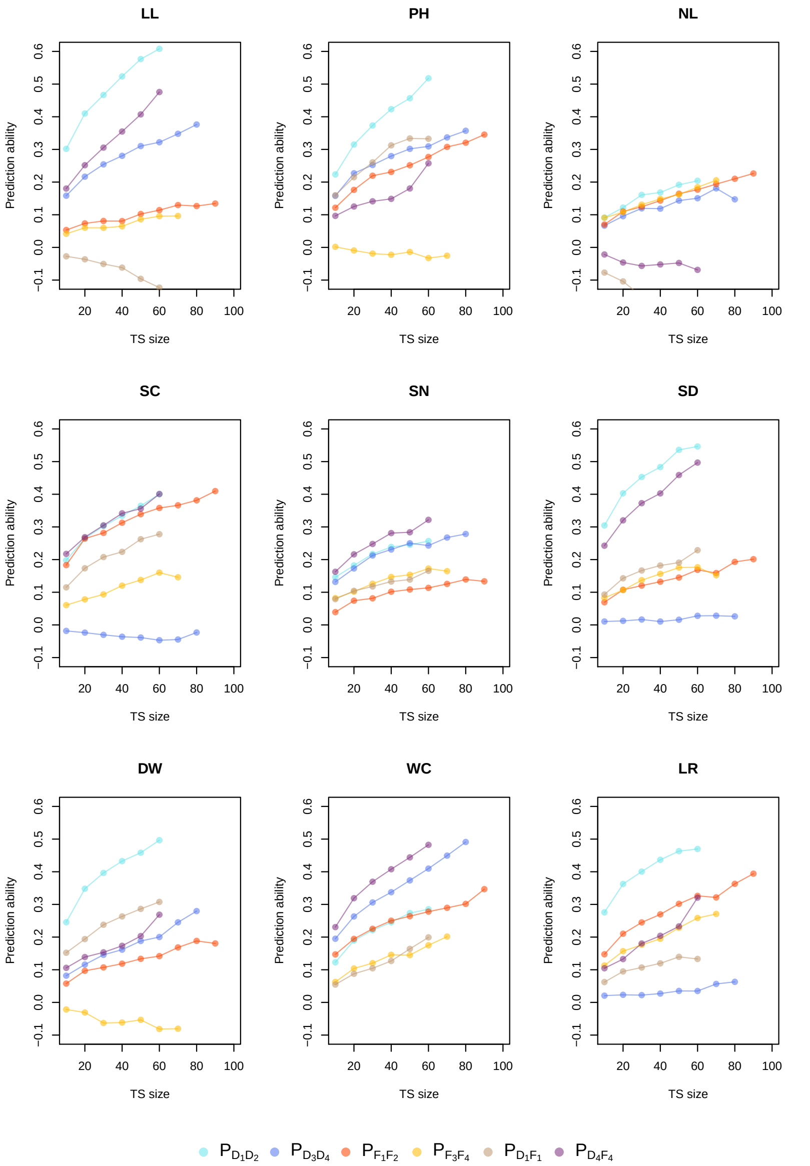

Supplementary Figure 15: Within-population prediction abilities r_{pop,BSP_w} for HSI for different training set (TS) sizes for each population based on $RAD_{482\text{-}GP:0.98}$ and the M_A model.

REFERENCES

[1] Danecek, P., Auton, A., Abecasis, G., Albers, C. A., Banks, E., DePristo, M. A., Handsaker, R. E., Lunter, G., Marth, G. T., Sherry, S. T., McVean, G., and Durbin, R. *Bioinformatics* **27**(15), 2156–2158 (2011).

[2] Li, H. *Bioinformatics* **27**(21), 2987–2993 (2011).

[3] Frey, F. P., Urbany, C., Hüttel, B., Reinhardt, R., and Stich, B. *BMC genomics* **16**(1), 123 (2015).

[4] Frey, F. P., Presterl, T., Lecoq, P., Orlik, A., and Stich, B. *Theoretical and Applied Genetics* **129**(5), 945–961 (2016).
